# Finding New Cell Wall Regulatory Genes in *Populus trichocarpa* Using Multiple Lines of Evidence

**DOI:** 10.3389/fpls.2019.01249

**Published:** 2019-10-08

**Authors:** Anna Furches, David Kainer, Deborah Weighill, Annabel Large, Piet Jones, Angelica M. Walker, Jonathon Romero, Joao Gabriel Felipe Machado Gazolla, Wayne Joubert, Manesh Shah, Jared Streich, Priya Ranjan, Jeremy Schmutz, Avinash Sreedasyam, David Macaya-Sanz, Nan Zhao, Madhavi Z. Martin, Xiaolan Rao, Richard A. Dixon, Stephen DiFazio, Timothy J. Tschaplinski, Jin-Gui Chen, Gerald A. Tuskan, Daniel Jacobson

**Affiliations:** ^1^Biosciences Division, and The Center for Bioenergy Innovation, Oak Ridge National Laboratory, Oak Ridge, TN, United States; ^2^The Bredesen Center for Interdisciplinary Research and Graduate Education, University of Tennessee, Knoxville, TN, United States; ^3^Oak Ridge Associated Universities (ORAU), Oak Ridge, TN, United States; ^4^Department of Chemical and Biomolecular Engineering, University of Tennessee, Knoxville, TN, United States; ^5^Department of Computer Science, Johns Hopkins University, Baltimore, MD, United States; ^6^Department of Biology, Johns Hopkins University, Baltimore, MD, United States; ^7^Oak Ridge Leadership Computing Facility, Oak Ridge National Laboratory, Oak Ridge, TN, United States; ^8^Department of Plant Sciences, The University of Tennessee Institute of Agriculture, University of Tennessee, Knoxville, TN, United States; ^9^Joint Genome Institute, Walnut Creek, CA, United States; ^10^HudsonAlpha Institute for Biotechnology, Huntsville, AL, United States; ^11^Department of Biology, West Virginia University, Morgantown, WV, United States; ^12^BioDiscovery Institute and Department of Biological Sciences, University of North Texas, Denton, TX, United States

**Keywords:** lines of evidence, cell wall, regulation, Genome Wide Association Study, candidate gene identification, network analysis, multi-omic, *Populus trichocarpa*

## Abstract

Understanding the regulatory network controlling cell wall biosynthesis is of great interest in *Populus trichocarpa*, both because of its status as a model woody perennial and its importance for lignocellulosic products. We searched for genes with putatively unknown roles in regulating cell wall biosynthesis using an extended network-based Lines of Evidence (LOE) pipeline to combine multiple omics data sets in *P. trichocarpa*, including gene coexpression, gene comethylation, population level pairwise SNP correlations, and two distinct SNP-metabolite Genome Wide Association Study (GWAS) layers. By incorporating validation, ranking, and filtering approaches we produced a list of nine high priority gene candidates for involvement in the regulation of cell wall biosynthesis. We subsequently performed a detailed investigation of candidate gene GROWTH-REGULATING FACTOR 9 (*PtGRF9*). To investigate the role of *PtGRF9* in regulating cell wall biosynthesis, we assessed the genome-wide connections of *PtGRF9* and a paralog across data layers with functional enrichment analyses, predictive transcription factor binding site analysis, and an independent comparison to eQTN data. Our findings indicate that PtGRF9 likely affects the cell wall by directly repressing genes involved in cell wall biosynthesis, such as *PtCCoAOMT* and *PtMYB.41*, and indirectly by regulating homeobox genes. Furthermore, evidence suggests that *PtGRF9* paralogs may act as transcriptional co-regulators that direct the global energy usage of the plant. Using our extended pipeline, we show multiple lines of evidence implicating the involvement of these genes in cell wall regulatory functions and demonstrate the value of this method for prioritizing candidate genes for experimental validation.

## Introduction

The biosynthesis and regulation of the plant cell wall has been the subject of a large body of research due to the industrial importance of lignocellulosic biomass, as well as the role of the cell wall in the function of other plant biological systems such as stress response, inter-cellular transport, and disease resistance. For industrially cultivated genera such as *Populus*, the primary cell wall constituents (i.e., cellulose, lignin, hemicellulose) provide feedstock for downstream products including biofuel, lumber, paper, and advanced lignin products (Sannigrahi et al., 2010; Porth et al., 2013). There is therefore broad interest in understanding the mechanisms that regulate the biosynthesis and modification of the cell wall, both from a yield and composition perspective.

A great variety of biopolymers are synthesized and incorporated into the primary and secondary cell wall, often in response to biotic and abiotic stress, nutrient availability, and developmental and temporal switches, all of which govern the macro-scale form of the plant. A highly complex network of genetic regulation has evolved to control the rate of biosynthesis of cell wall polymers, their intrinsic monomer composition, their transport to and subsequent deposition in the cell wall, and the expansion of the wall under changing intra-cellular conditions. In the model plant *Arabidopsis thaliana*, Bischoff et al. (2010) estimated that over 1,000 genes encode proteins related to the cell wall, while Cai et al. (2014) predicted a number closer to 3,000 based on clustering of gene co-expression (Bischoff et al., 2010; Cai et al., 2014). Furthermore, Taylor-Teeples et al. (2015) tested a library of 1,664 transcription factors in *A. thaliana* for interaction with the promoter regions of cell wall biosynthesis genes and found 413 such interactions in root vascular tissue alone (Taylor-Teeples et al., 2015). Studies such as these highlight the immense complexity involved in cell wall regulation, much of which is still to be elucidated.

Due to poplar’s status as a model woody plant and its importance for lignocellulosic products, many studies have investigated the regulatory network of the cell wall and its components in *Populus* species or in multiple genera in combination with *Populus* (Ohtani et al., 2011; Puzey et al., 2012; Lu et al., 2013; Porth et al., 2013; Yu et al., 2013; Ko et al., 2014; Wang et al., 2014; Zhong and Ye, 2014; Muchero et al., 2015; Lin et al., 2017; Shi et al., 2017; Xie et al., 2018a). Many of these studies have focused either on characterizing *Populus* homologs of genes that have been shown to have an effect on the cell wall chemistry or plant growth traits in mutant Arabidopsis lines, or perhaps were shown to be differentially expressed in comparisons of low and high growth genotypes. However, exploring the regulatory network controlling the cell wall in order to find new functional mechanisms is a challenging task due to the number of genes involved, extensive functional redundancy, and the multitude of transcriptional feedback loops. Such complex genetic architecture has contributed to the view that many quantitative traits are actually “omnigenic” (Boyle et al., 2017), such that virtually any expressed gene has a non-zero effect on the core biosynthetic genes at one or more transcriptional, post-transcriptional, post-translational, signaling or protein-protein interaction levels. Fisher (1919) predicted that rather than a few core genes in biosynthetic pathways, the major portion of heritability is explained by a large number of loci across the entire genome that contribute small portions of the trait heritability. Under this omnigenic model, network-theory-based methods provide an elegant approach for mining omics datasets for regulatory relationships. Any biological entity (SNP, gene, protein, metabolite, etc.) can be modeled as a node and any relationship between those entities (association, co-expression, correlation, binding) can be modeled as an edge.

The network approach has been used in several studies of cell wall regulation to date, often focusing on finding clusters of genes that co-express with each other in certain tissues, thus finding putative functional units or networks. For example, Cai et al. (2014) performed co-expression network clustering in *Populus* and found major sub-clusters enriched for primary cell wall or secondary cell wall genes. Taylor-Teeples et al. (2015) produced networks based on *A. thaliana* transcription factors and their target binding sites, providing an expanded view of the multi-tiered regulatory system with respect to secondary cell wall (SCW) biosynthesis and xylem development. Yang et al. (2011) used 121 A*. thaliana* cell wall genes obtained from text mining followed by co-expression neighbor analysis to identify 694 A*. thaliana* genes and their 817 *Populus* orthologs as candidate genes for involvement in cell wall functions. Alejandro et al. (2012) identified the ABCG29 genes as transporting monolignol to the cell wall in *A. thaliana* by first analyzing co-expression networks followed by expression and functional analyses. These methods often produce an extensive list of candidate genes but with little more to support their involvement in cell wall regulation than the clustering or enrichment evidence.

Multi-omic approaches have also been performed, which include more data types to identify candidate genes. Porth et al. (2013) used a network-based multi-omic approach to find relationships between SNP, gene expression, and wood phenotype data from *P. trichocarpa*. They constructed six phenotypic-centric networks to identify genes that most influenced the expression of their related phenotype. From this study, they were able to identify candidate genes potentially related to cell wall biogenesis. Mizrachi et al. (2017) used a network-based approach to integrate known gene interactions and eQTN data in the form of a connectivity matrix with gene expression data through matrix multiplication in order to identify genes involved in lignin-related traits.

**Figure 1:**
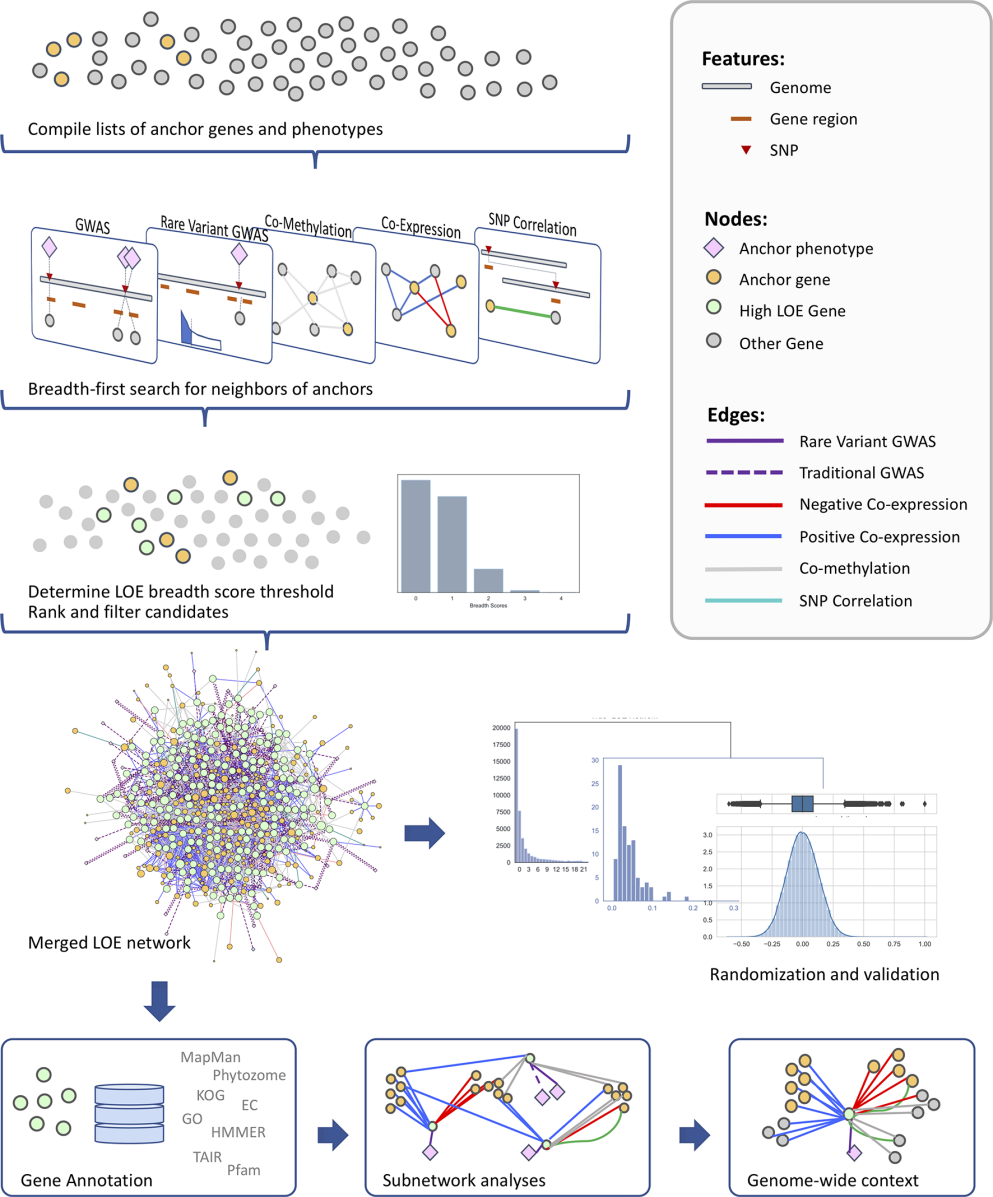
Overview of the method for identifying new candidate genes involved in cell wall regulation through data layering and calculation of LOE scores.

The use of multiple layers of omics data in the identification of candidate genes related to a particular phenotype provides an increased level of confidence and context surrounding the new candidate genes. In this study, we use an extended lines of evidence (LOE) pipeline for jointly mining multiple data layers to produce a curated short list of new candidate genes putatively involved in the regulation of cell-wall-related functions (**Figure 1**). We use an extensive set of “anchor” genes with documented roles in cell wall biosynthetic and regulatory processes and anchor metabolomic phenotypes measured in a Genome Wide Association Study (GWAS) population of *P. trichocarpa*. Multi-omic data layers (coexpression, comethylation, pairwise SNP correlation, and two SNP-metabolite GWAS data sets) are probed to find all genes in the genome with network connectivity to the anchor set. A score is calculated for each gene with regards to the amount of evidence that the gene is involved in cell wall regulation and other cell-wall-related processes. The resulting merged LOE network of candidate genes is then subjected to validation, ranking, and filtering methods, as well as post-hoc analyses. The result is a set of 330 high-ranking candidate genes, which we then filter to a subset of regulatory genes not previously discussed in the context of the cell wall biosynthesis.

## Materials and Methods

This study makes use of various data accumulated for *P. trichocarpa* that have been used in previous investigations, including SNP data from a GWAS population, foliar metabolites measured in this GWAS population, and DNA methylation data across 10 different *P. trichocarpa* tissues (Vining et al., 2012), as well as the *P. trichocarpa* DOE Joint Genome Institute Plant Gene Atlas (Sreedasyam et al, unpublished data; available from phytozome.jgi.doe.gov). Each data set was considered as a separate layer for this study, and integrated though the use of LOE scores. Below, the various layers are described as well as the network analysis methods used to merge layers and identify genes with high connectivity to cell wall systems.

### Phenotypes

We made use of metabolite data previously obtained from leaf tissue and analyzed using GC-MS. Details can be found in (Tschaplinski et al., 2012; Li et al., 2012b; Weighill et al., 2018). To prevent spurious associations, we examined each phenotype for the presence of outliers using Median Absolute Deviation (MAD). If a sample’s phenotype was more than six MADs from the population median it was removed from the GWAS for that phenotype.

### Genotypes

SNP-based variant data (see DOI 10.13139/OLCF/1411410) were obtained from https://bioenergycenter.org/besc/gwas/ and SNPs were filtered to the top 90% tranche (PASS SNPs) and call rate ≥ 0.5 using Plink (Purcell et al., 2007) and VCFtools (Danecek et al., 2011).

### Genome Wide Association Layer

GWAS was performed using a linear mixed model (LMM), implemented in EMMAX (Kang et al., 2010) and leveraging ADIOS v1.13 (Lofstead et al., 2008) for scaling, to estimate the additive effect of each SNP while accounting for population structure and cryptic relatedness between samples. The tested SNPs excluded those with minor allele frequency (MAF) < 0.01, and those with a population call rate above 0.75. In addition, we used linkage disequilibrium (LD) pruning on the main set of SNPs to produce a set of independent SNPs for estimating the genomic relationship matrix, used in the LMM. The resulting p-values were corrected for multi-hypotheses bias by applying the Benjamini-Hochberg approach (Benjamini and Hochberg, 1995) with a false-discovery rate (FDR) cutoff of 0.1.

### Rare Variant GWAS Layer

While the GWAS Linear Mixed Model (LMM) tested common and less common SNPs (MAF ≥0.01) individually for significance, rarer SNPs were tested regionally in a joint fashion. Rare SNPs (MAF <0.01) located within a given gene, or in the gene’s 2-kb upstream and downstream flanking regions, were grouped as a region defined by that gene. RVtest (Zhan et al., 2016) was then used to apply the Sequence Kernel Association Test (SKAT) to each of the 41,335 regions defined from *P. trichocarpa* v3.0 annotations. SKAT tests each SNP in the region individually with an LMM and then forms a combined region score where each component SNP is weighted according to its MAF. Weights were drawn from a beta distribution with default shape parameters (1, 25), which produced a single P-value for the significance of association of each region, which were corrected for multiple testing with an FDR of 0.1.

### Co-Expression Layer

A *P. trichocarpa* gene co-expression network was constructed as described in Weighill et al. (2018). RNA-seq reads from the *P. trichocarpa* DOE Joint Genome Institute Plant Gene Atlas (Sreedasyam et al., unpublished data; available from phytozome.jgi.doe.gov; see **Supplementary Table S1** for sample information) were trimmed using Skewer (Jiang et al., 2014), aligned to the version 3.0 *P. trichocarpa* reference (Tuskan et al., 2006) using Star (Dobin et al., 2013), and TPM (transcripts per million) values calculated for each gene and each sample. Star mapping was performed using the “–quantMode GeneCounts” option, which directs the program to count the number of reads per gene while performing the mapping. A read is counted if it overlaps one and only one gene. Both ends of the paired-end read are checked for overlaps. The counts coincide with those produced by htseq-count with default parameters. We then calculated the Spearman correlation coefficient between the expression profiles of all pairs of genes using the mcxarray package (Van Dongen, 2008) available from https://micans.org/mcl/index.html. An absolute threshold of 0.85 was applied in order to keep only those gene-gene pairs with strong co-expression.

### Co-Methylation Layer

A *P. trichocarpa* gene co-methylation network was constructed as described in Weighill et al. (2018). MEDIP-Seq reads from the study by Vining et al. (2012) mapped to the *P. trichocarpa* V3 genome assembly, were obtained from Phytozome (Goodstein et al., 2011; Vining et al., 2012). The number of reads that mapped to each gene for each sample was determined using htseq-count (Anders et al., 2015). These counts were then converted to TPM values for each gene and each sample. Spearman correlation coefficients between the co-methylation profiles of all pairs of genes were then calculated in a manner similar to the co-expression layer, followed by an absolute threshold of 0.95.

### Custom Correlation Coefficient Layer

After filtering the SNP set to remove those with MAF <0.01, the custom correlation coefficient (CCC) (Climer et al., 2014) between all pairs of remaining SNPs were calculated using a Parallel GPU implementation of the CCC (Joubert et al., 2017). In order to minimize correlation among SNPs due to linkage disequilibrium, only correlations from SNP pairs greater than 10 kb apart and with a CCC ≥0.7 were retained. SNPs were then mapped to the genes in which they were located, resulting in gene-gene correlations. Significantly correlated SNPs represent co-segregating and interacting cellular components.

### Lines of Evidence Scoring and Network Analysis

The LOE method calculates a score for every gene in the genome by quantifying the connectivity of a given gene to anchor genes/phenotypes from the system of interest. Each data layer described above provides one possible line of evidence. For example, if Gene A co-expresses with one or more cell wall anchor genes, then this is counted as one line of evidence for Gene A’s involvement in the cell wall. A list of 295 anchor genes was compiled from the literature (Hao and Mohnen, 2014; Zhong and Ye, 2014; Nakano et al., 2015; Liu et al., 2017; Rao and Dixon, 2018) (**Supplementary Table S2**). Metabolites that affect cell wall development and composition, such as sugar substrates, lignin precursors, and lignin competitors, were also selected for use as cell wall anchor phenotypes (**Supplementary Table S3**).

To calculate LOE scores for each gene in the *P. trichocarpa* genome, each data layer was represented as a network. Each layer consisted of a list of source entities (cell wall anchor genes and phenotypes, or “anchor nodes”), target entities (potential candidate genes, or “target nodes”), and interactions between them (correlations/associations, or “edges”). From each layer’s network, a breadth-first search was used to extract the neighbors of anchor nodes, resulting in a “one-hop” (“1-hop”) network for each layer. LOE scores were calculated as per Weighill et al. (2018). Briefly, the LOE breadth score for a gene is the count of the different layers in which that gene has connections to anchor genes/phenotypes. An LOE depth score—the count of all connections to anchor genes/phenotypes across all data layers—was also calculated for each gene. After scoring, the 1-hop networks from all layers were thresholded based on the distribution of LOE breadth scores, then merged to form the LOE network containing cell wall anchor genes and phenotypes and all genes connected to them *via* one or more layers (“high LOE genes”). All genes in the merged LOE network were ranked based upon breadth and depth scores and genes with previously documented cell-wall-related roles were removed. Networks were visualized and manipulated with Cytoscape 3.6.1 (Shannon et al., 2003).

### Gene Annotation, Functional Enrichment, and Expression Analyses

Functional annotations for *P. trichocarpa* genes were obtained from JGI Phytozome 12 (Goodstein et al., 2011) and MapMan using the Mercator tool (Lohse et al., 2014). A number of high LOE genes were not annotated in MapMan or Phytozome. To better understand the potential functions of those genes, protein sequences were extracted from the *P. trichocarpa* v3.1 primary transcript sequence (Tuskan et al., 2006) available from Phytozome and analyzed using HMMER v3.1b2 (Eddy, 1998) to annotate both Pfam v31.0 (Punta et al., 2011) and TIGRfam v15.0 (Haft et al., 2001) domains. Domains were thresholded using an independent E-value of 0.001. GO-term enrichment was performed on selected sets of genes using the BinGO Cytoscape app (Maere et al., 2005) using the Hypergeometric Test as well as Benjamini & Hochberg False Discovery Rate Correction at a significance level of 0.1.

A clustered heatmap of gene expression data was created using the Python (v3.6.2) package seaborn (v0.8.0; https://seaborn.pydata.org/index.html). Prior to analysis, six samples were removed from the data set that were outliers relative to their tissue type and treatment subgroups. Gene expression was normalized across tissues and genes were clustered using a Euclidean distance metric and Ward clustering method.

To assess orthology for a subset of genes during post-hoc analyses in Section 4.4.1, amino acid sequences containing characteristic PFAM domains (http://pfam.xfam.org/) were obtained from UniProt (www.uniprot.org; KNOXI: PF03790 per Mukherjee et al., 2009; POX/BELL: PF07526 per Bellaoui et al., 2001) and reciprocal BLASTp searches were performed against *P. trichocarpa* and *A. thaliana* genomes using NCBI’s BLAST (https://blast.ncbi.nlm.nih.gov) with default settings.

### Network Validation

#### Randomizations of Expression and Methylation Data

We assessed whether our coexpression and comethylation networks contain greater biological signal than random networks by performing analyses on multiple randomized expression and methylation datasets. First we generated 100 randomized gene expression data sets by shuffling TPM values within genes across tissues, thereby preserving the observed range of expression for each gene but destroying the associations with tissue samples. We then generated a Spearman coexpression matrix for each random dataset and randomly subsampled 100,000 correlation values from each, resulting in a total pool of 10,000,000 random coexpression samples. We then collected 10,000,000 random subsamples from our observed coexpression data set and compared the distributions of our observed values to those of the shuffled data sets using the Wilcoxon rank-sum test using the Python package SciPy stats module (docs.scipy.org/doc/scipy/reference/generated/scipy.stats.ranksums.html). We also performed this method with the comethylation data layer.

#### Functional Validation of LOE Network

To assess whether our observed LOE network captured a greater amount of biological function than random networks, we intersected the observed network as well as 100 randomized LOE networks with a GO-term functional network. We first constructed a functional network from GO Biological Process terms whereby genes that share GO terms are connected and are more likely to share biological function than unconnected genes. GO annotations for *P. trichocarpa* genes were obtained from PlantRegMap (Jin et al., 2017) and we removed any term present in over 1000 genes to avoid generating an overly dense network from highly generic functions. Furthermore, we weighted edges with a score inversely proportional to the number of genes with that GO term, such that between genes due to rarer GO terms were considered more functionally valuable than edges due to broader GO terms. If two genes shared multiple GO terms then we retained only the higher scoring edge. We then generated 100 randomized networks for each input data layer by holding anchor nodes and edges constant and replacing their 1-hop neighbors with gene labels randomly drawn from the genome, thereby ensuring that the size and structure of the randomized networks were comparable to the LOE input networks. For each set of random networks (consisting of one randomized network of each type: comethylation, coexpression, SNP correlation, traditional metabolite-GWAS, and rare variant metabolite-GWAS), LOE scoring and thresholding was performed. Each merged LOE network was then intersected with a GO-term functional network and an intersect score was recorded. The intersect score is calculated by summing the values of the GO-term network edges that are also present in the LOE scored network. We then compared the intersect score of our observed LOE network to the distribution of randomized network intersect scores.

#### Expression Quantitative Trait Networks

We utilized eQTN data as an independent line of evidence for investigating the putative regulatory roles of the *PtGRF9* paralogs. RNAseq sequencing data from (Zhang et al., 2018) were obtained from the NCBI SRA database (SRA numbers: SRP097016–SRP097036; www.ncbi.nlm.nih.gov/sra). Reads were aligned to the *Populus trichocarpa* v.3.0 reference (Tuskan et al., 2006), using STAR (Dobin et al., 2013). Transcript per million (TPM) counts were then obtained for each genotype, resulting in a genotype-transcript matrix. For each gene transcript we determined outlier values, masking TPM values that exceeded a median absolute deviation from the non-zero median threshold of 5.0. Transcripts that had a non-outlier observed TPM value in more than 20% of the population were retained for further analysis. These expression profiles were then used as phenotypes in a GWAS, using EMMAX (Kang et al., 2010). Single nucleotide polymorphisms (SNPs) data, for the same population of *P. trichocarpa* genotypes, was obtained from (DOI 10.13139/OLCF/1411410). The SNPs were processed using VCFTOOLS (Danecek et al., 2011) and PLINK (Purcell et al., 2007), selecting for the 90% tranche and a minor allele frequency of 0.01. A hierarchical approach (Peterson et al., 2016) was used to correct for multiple hypotheses bias associated with the number of phenotypes. The procedure involved two rounds of false discovery rate (FDR) corrections, the initial using the Benjamini-Hochberg (Benjamini and Hochberg, 1995) procedure (q1 < 0.1), followed by the Gavrilov-Benjamini-Sarkar stepdown approach (Gavrilov et al., 2009) (q2 < 5.1e-4). SNP to phenotype association that passed the respective thresholds were determined to be statistically significant. 1-hop eQTN networks were then created around the *PtGRF9* paralogs.

## Results and Discussion

### Evaluation of Expression and Methylation Data

The Wilcoxon rank-sum test was used to determine whether the distribution of correlation values differed between our observed data set and values from randomized datasets (**Supplementary Figure S1**). For both the expression and methylation data sets, the observed distributions were significantly different to random (p < 0.01 for both data types). Our coexpression data layer was thresholded to exclude correlation values below 0.85, resulting in 16,122 values (0.19%) being retained. In our shuffled data set, only 45 values (or 5.25e-04%) were above the 0.85 threshold. Our comethylation data layer was thresholded to exclude correlation values below 0.95, resulting in 87,458 values (0.88%) being retained. In our shuffled data set, only 1,090 values (0.01%) were above the 0.95 threshold.

### Construction of LOE Network

The LOE method was used to identify new candidate genes involved in regulating the cell wall in *P. trichocarpa* by jointly probing five different omics data layers. LOE depth scores were calculated for each gene, indicating the number of lines of evidence within each layer connecting that gene to an input set of cell wall anchor genes and metabolites. An LOE breadth score was also calculated for each gene, indicating the number of types of lines of evidence that connected the gene to input cell-wall-related targets. A merged LOE network was created after determining an appropriate LOE breadth score threshold and taking the union of all thresholded input networks. Threshold criteria dictated that candidate genes have a significant association with one or more metabolites in either the traditional or rare variant data layers as well as a total breadth score of three. We required a minimum of one GWAS association for retention in the merged network because metabolite-GWAS associations represent a measurable cell wall phenotype. A breadth score of three was selected in order to prioritize a small set of genes having strong evidence for involvement in cell-wall-related processes, and the distribution of breadth scores exhibits an inflection point at three (**Supplementary Figure S2A**). These criteria identified a list of 315 “high LOE genes” as potential candidates for involvement in cell-wall-related functions. Seven high LOE genes had a breadth score of four and 308 had a breadth score of three (**Supplementary Figure S2B**). Overall, high LOE genes were from a variety of functional categories (**Supplementary Figure S3**) and 80 of these genes were annotated with potential regulatory functions (**Supplementary Table S4**).

#### Candidate Gene Ranking

To prioritize candidates, we created three ranked tiers to which high LOE genes were assigned (Tier 1 is the highest priority, Tier 3 is the lowest priority). Genes were ranked by 1) breadth score and 2) total depth score minus co-methylation depth score. While our co-expression data vectors contain 64 data points per gene (64 tissues and experimental conditions), our co-methylation data vectors contain only 10 data points per gene (10 tissues and experimental conditions), resulting in an increased probability for spurious correlations in the co-methylation data layer. While the distribution of comethylation correlation values (**Supplementary Figure S1**) was significantly different than random, the shape of the distribution suggests a conservative approach is warranted. In order to avoid upwardly biasing gene rankings, co-methylation data was included in the first stage of the ranking process (overall rank by breadth score) but excluded from the second stage of the ranking process (ranking within breadth score bins by depth score). Genes with an LOE Breadth score of four were included in Tier 1 by default (seven genes). In addition, genes with an LOE Breadth score of three and total depth minus comethylation depth scores of five or greater were included in Tier 1, resulting assignment of 45 genes. Thirty-two genes were assigned to Tier 2 based on a total depth minus comethylation depth score of four. The remaining 238 high LOE genes had total depth minus comethylation depth scores of three or less and were assigned to Tier 3.

#### Functional Validation of LOE Network

**Figure 2:**
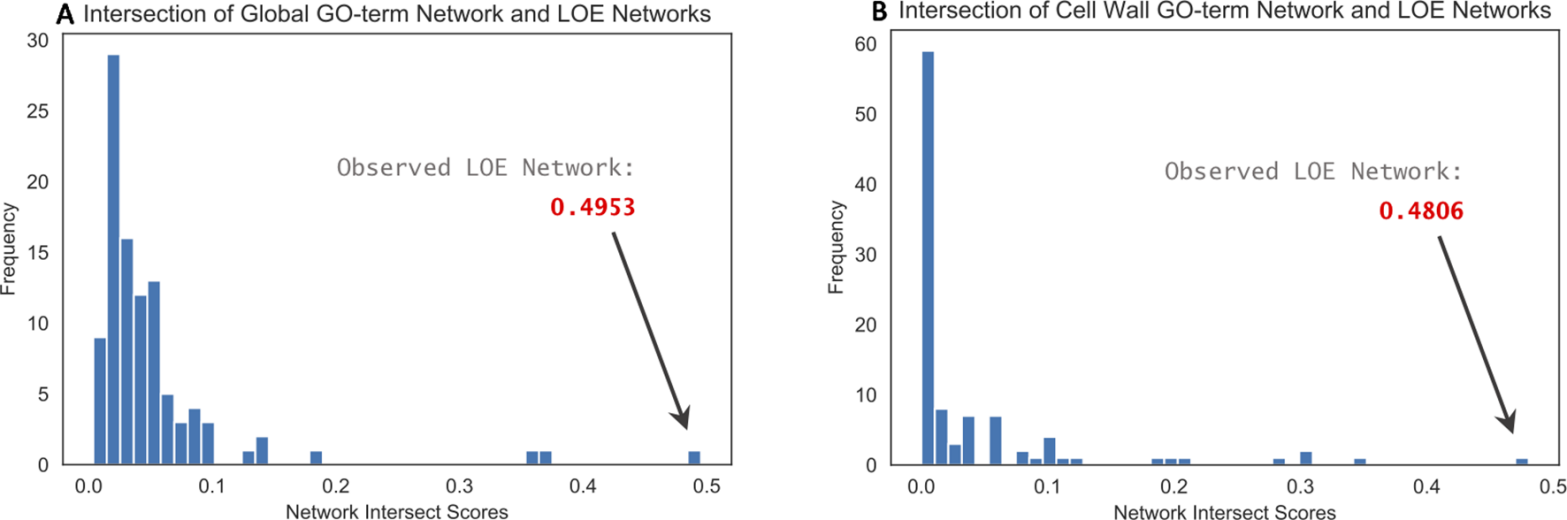
Histograms of network intersect scores calculated by intersecting the observed and randomized LOE networks with GO-term functional networks. **(A)** Intersection with the global GO-term functional network resulted in a score of 0.4953 for the observed LOE network; intersect scores for randomized networks were ≤0.3701. **(B)** Intersection with the cell wall-specific GO-term functional network resulted in a score of 0.4806 for the observed LOE network; intersect scores for randomized networks were ≤0.3470.

Intersection of the observed thresholded LOE network with the global GO-term functional network resulted in an intersect score of 0.4953, whereas intersect scores for the 100 randomized LOE networks (also thresholded) ranged from 0 to 0.3701 (**Figure 2A**). Intersection of the observed LOE network with the cell wall-specific GO-term network resulted in a score of 0.4806; intersect scores for the 100 randomized networks ranged from 0 to 0.3470 (**Figure 2B**). These results imply that our observed LOE network captures a greater amount of biological signal than the randomized LOE networks.

#### Literature Evidence

**Figure 3:**
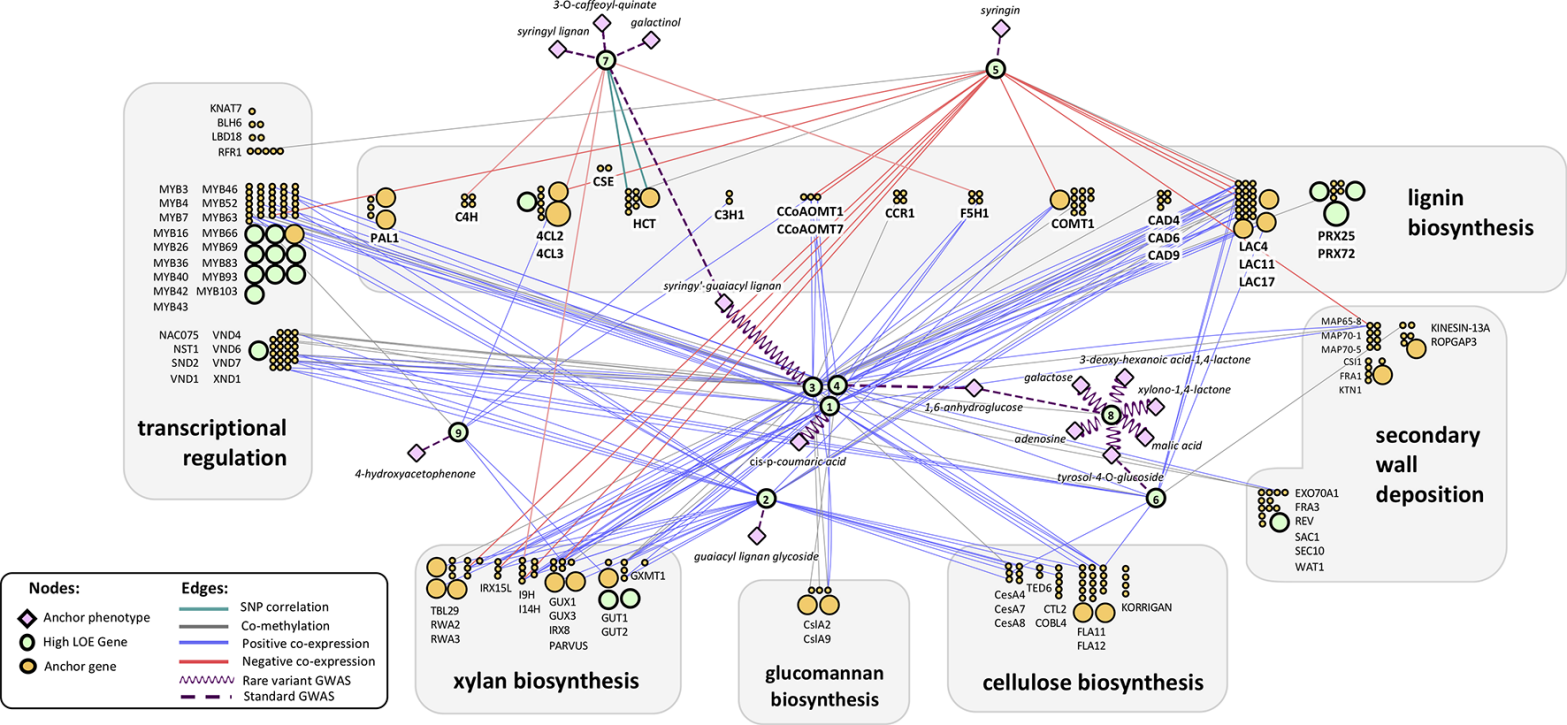
Tier 1 high LOE genes with regulatory annotations in the context of the LOE network arranged to highlight each gene’s connection to lignin/cell wall biosynthesis pathways. Orange and green circles represent cell wall anchor genes and high LOE genes, respectively. Numbers within high LOE genes (green circles) indicate an entry within **Table 1**. Green circles that do not contain numbers represent a subset of the high LOE genes that were filtered from the final results set due to having prior evidence of cell-wall-related functions in the literature. The size of circles corresponds to their LOE breadth score. Gene symbols are Arabidopsis Best-hit matches.

Recovering genes for which cell-wall-related functions have been previously reported is an important internal validation for the LOE method. We performed an extensive literature review to find evidence of previously validated genes in our results set. Forty-four genes were recovered with previous validation regarding cell-wall-related functions in *P. trichocarpa*, Arabidopsis, or other plant species and for which there is evidence of orthology in *P. trichocarpa* (see **Supplementary Table S5**). Fifteen of these high LOE genes were also in our anchor gene list. Genes with prior evidence of cell-wall-related functions were removed from our merged LOE network in order to present researchers with “new” candidate genes: 14 from Tier 1, four from Tier 2, and 11 from Tier 3; 17 of these genes are represented in **Figure 3**. However, the literature review process was not as thorough for Tiers 2 or 3, thus it is possible that some of the remaining genes in these tiers have prior connections to cell wall processes. The full ranked and filtered high LOE gene list can be found in **Supplementary Table 6**. For the remainder of the manuscript, we focus on Tier 1 genes.

A notable example of a high LOE gene with prior evidence of a cell wall regulatory role is IQ-domain 10 calcium-signaling gene *PtIQD10* (Potri.011G096500). *PtIQD10* has a breadth score of three and a depth score of 48, including rare variant metabolite-GWAS associations with syringin, coniferin, and xylulose, and significant coexpression and comethylation with 41 cell wall anchor genes (**Supplementary Table S5**, **Supplementary Figure S4**, and **Supplementary Table S6**). The Arabidopsis ortholog *AtIQD10* (AT3G15050; orthology with *PtIQD10* and *P. deltoides* *PdIQD10* supported by phylogenetic analysis in Badmi et al., 2018) is differentially expressed in Arabidopsis lines overexpressing the transcription factor SECONDARY WALL-ASSOCIATED NAC DOMAIN PROTEIN 2 (*AtSND2*) (Hussey et al., 2011). Hussey et al. (2011) hypothesize *AtIQD10* activates AtSND1 NAC, followed by activation of SND2, MYBs, and cell wall polymerization functions. Consistent with this model, orthologs of these genes are present in the *PtIQD10* one-hop neighborhood (**Supplementary Figure S4**). Additional evidence has recently been observed in *P. trichocarpa* congeners. An ortholog of *PtIQD10* in the *P. alba* x *P. glandulosa* hybrid “84k” is differentially expressed during the transition between primary and secondary growth phases in stems (Li et al., 2017). In addition, *P. deltoides* ortholog *PdIQD10* has higher expression levels in tension-stressed xylem tissues and secondary walled cells, and RNAi repression of *PdIQD10* results in altered phenotypes such as increased cellulose, wall glucose content, plant height, stem count, and stem density (Badmi et al., 2018; Macaya-Sanz et al., 2017). *PdIQD10* is coexpressed with secondary cell wall related genes such as *SUSY*, *CESAs*, and *KOR* (Badmi et al., 2018), orthologs of which are present in our PtIQD10 subnetwork (Potri.018G103900 cellulose synthase/*PdCesA7-B*/*AtCESA7* and Potri.004G059600 *PtCESA.2*/*PdCESA8-B*/*AtCESA8*; see **Supplementary Table S7** for the *PtIQD10* one-hop subnetwork node information for **Supplementary Figure S4**).

In another example of a high LOE gene with prior evidence of a cell-wall-related role, Porth et al. (2013) found that a SNP in an exostosin family protein gene (Potri.019G044600) involved in xylogalacturonan biosynthesis was correlated with xylose (hemicellulose) content. In yet another example, Pomiès et al. (2017) found a berberine bridge enzyme gene (Potri.011G161500) with orthology to *AtEDA28/MEE23* (AT2G34790, shown to play a role in lignin monolignol metabolism) was highly up-regulated 72 h after mechanical perturbation of stems as plants modified cell wall properties in response. Another example with growing evidence of cell-wall-related regulatory functions is MADS-box transcription factor *PtAGL12* (Du et al., 2009; Du et al., 2011; Weighill et al., 2018; see **Supplementary Text S1**, **Supplementary Figure S5**, **Supplementary Table S8**, and **Supplementary Figure S6** for additional evidence regarding the putative role of *PtAGL12* in regulating cell wall biosynthesis).

### Tier 1: Highest Priority Candidates for Cell Wall Regulation

Tier 1 genes have the strongest evidence of involvement in cell wall related processes (**Table 1**). Of these, nine genes had regulatory annotations (via MapMan, www.arabidopsis.org, or PFAM; see **Supplementary Table S4** for categories considered regulatory). While the remaining 21 genes did not have regulatory annotations, our results suggest they play a role in cell wall biosynthesis.

**Table 1:** Tier 1 high LOE genes. See **Supplementary Table S9** for additional score and annotation information.

Node #	Gene ID	Arabidopsis gene/domain symbol	Description	Arabidopsis ortholog
**Regulatory genes**				
**1**	Potri.008G112300		DNA glycosylase superfamily protein	AT1G13635.2
**2**	Potri.001G216000	EAR1	ENHANCER OF ABA CO-RECEPTOR 1	AT5G22090.1
**3**	Potri.013G060500	ATCRT1	RING/U-box superfamily protein	AT5G56340.1
**4**	Potri.013G156300	Shisa	Wnt and FGF inhibitory regulator	
**5**	Potri.015G006200	AtGRF7, GRF7	growth-regulating factor 7	AT5G53660.1
**6**	Potri.017G053000	AMC1, ATMC1, ATMCPB1	metacaspase 1	AT1G02170.1
**7**	Potri.018G105600	YbaB_DNA_bd	YbaB/EbfC DNA-binding family	AT2G24020.1
**8**	Potri.013G093800		eukaryotic translation initiation factor SUI1 family protein	AT1G71350.1
**9**	Potri.010G072700		RING/U-box superfamily protein	AT5G43420.1
**Other genes**				
	Potri.004G085400	ATGLN1;1, ATGSR1, GLN1;1, GSR 1	glutamine synthase clone R1	AT5G37600.1
	Potri.006G256000		Phox (PX) domain-containing protein	AT4G32160.1
	Potri.012G093800	ATNDPK2, NDPK1A, NDPK2	nucleoside diphosphate kinase 2	AT5G63310.1
	Potri.010G155600		Leucine-rich repeat transmembrane protein kinase	AT1G53440.1
	Potri.001G340400	SEO_N	SEO_N–Sieve element occlusion N-terminus	
	Potri.006G153300		N-acetylated-alpha-linked acidic dipeptidase (NAALAD)	AT5G19740.1
	Potri.008G156600	AST12, SULTR3;1	sulfate transporter 3;1	AT3G51895.1
	Potri.003G079900	AW: HRGP	hydroxyproline-rich glycoprotein family protein	AT4G16790.1
	Potri.T135500	CYCP4;1	cyclin p4;1	AT2G44740.1
	Potri.018G090300	AHA1, HA1, OST2, PMA	H(+)-ATPase 1	AT2G18960.1
	Potri.017G059300	SHM4	serine hydroxymethyltransferase 4	AT4G13930.1
	Potri.004G059900		Protein of unknown function DUF1685	AT2G42760.1
	Potri.016G115200	LHCB4.2	light harvesting complex photosystem II	AT3G08940.2
	Potri.015G063400	ATATH2, ATH2	ABC2 homolog 2	AT3G47740.1
	Potri.019G087700	ATSERK1, SERK1	somatic embryogenesis receptor-like kinase 1	AT1G71830.1
	Potri.007G027400		anti-muellerian hormone type-2 receptor	AT3G50685.1
	Potri.005G067000		Protein kinase protein with adenine nucleotide alpha hydrolases-like domain	AT1G77280.1
	Potri.001G352200	ATPUP10, PUP10	purine permease 10	AT4G18210.1
	Potri.011G142200	PSBR	photosystem II subunit R	AT1G79040.1
	Potri.006G060100	CRR6	chlororespiratory reduction 6	AT2G47910.1
	Potri.010G113700	FAB1C	FORMS APLOID AND BINUCLEATE CELLS 1C	AT1G71010.1

Among Tier 1 regulatory genes, there were a total of 18 metabolite-GWAS associations, 8 of which were rare variant hits (**Figure 3**). Potri.013G093800 (Arabidopsis homolog AT1G71350, a eukaryotic translation initiation factor SUI1 family protein) has the highest number of rare variant metabolite-GWAS associations (six) of any high LOE gene as well as the highest number of total combined GWAS edges (seven). Most Tier 1 regulatory genes share edges with cell wall anchor genes from multiple process categories (in **Figure 3**, gray boxes indicating functional groupings of cell wall anchor genes). On average, Tier 1 genes were connected by multiple edges to four different functional groups, suggesting that Tier 1 genes influence multiple aspects of cell wall biosynthesis. Furthermore, eight Tier 1 regulatory genes shared edges with anchor cell wall transcriptional regulation genes (**Figure 3**).

Notably, coexpression edges for Tier 1 regulatory genes were either strictly negative for a given gene, or strictly positive, perhaps hinting at the regulatory mechanism of each gene. Two Tier 1 regulatory genes (Potri.015G006200: GROWTH-REGULATING FACTOR 9/*PtGRF9* and Potri.018G105600: NUCLEOID-ASSOCIATED PROTEIN YBAB) were negatively coexpressed with cell wall genes and six were positively co-expressed with cell wall genes. The negatively coexpressed genes (Potri.015G006200, Potri.018G105600) did not share any neighbor nodes, however they are both connected to lignin and xylan biosynthesis genes. In contrast, positively coexpressed Tier 1 regulatory genes had a large overlap in neighbor cell wall anchor genes. The overlap was even more pronounced among Potri.008G112300, Potri.001G216000, Potri.013G060500, and Potri.013G156300 despite a complete lack of overlap among metabolite-GWAS edges or MAPMAN functional annotations (**Supplementary Table S9**).

We conducted an in-depth investigation into the Tier 1 regulatory gene *PtGRF9* (Potri.015G006200) to assess support for *PtGRF9* playing a regulatory role in cell wall biosynthesis.

### GROWTH-REGULATING FACTOR 9: Putative Master Regulator

The transcription factor gene GROWTH-REGULATING FACTOR 9 (*PtGRF9*/Potri.015G006200) had a breadth score of three and depth score of 17, including 13 negative coexpression edges (the highest negative coexpression depth score in our analysis). *PtGRF9* shared nine edges with lignin biosynthesis genes, four edges with xylan biosynthesis genes, two edges with transcriptional regulation genes, and one edge with a secondary cell wall deposition gene.

The *P. trichocarpa* genome annotation (v3.0; available on https://phytozome.jgi.doe.gov; Tuskan et al., 2006) indicates the best-hit Arabidopsis match for *PtGRF9* is AT5G53660 (growth-regulating factor 7, *AtGRF7*). To assess support for orthology, we performed reciprocal BLASTp searches of amino acid sequences containing the WRC (PF08879) and QLQ (PF08880) domains from *A. thaliana* and *P. trichocarpa* (obtained from UniProt; www.uniprot.org) and a phylogenetic analysis (see **Supplementary Figure S7**). Our results support an orthologous relationship between *PtGRF9* and *AtGRF7*, which is consistent with the phylogenetic analysis of Cao et al. (2016). While investigating support for orthology between *PtGRF9* and *AtGRF7*, we discovered a second *AtGRF7* ortholog in the *P. trichocarpa* genome (Potri.012G022600; hereafter, Potri.015G006200 is referred to as “*PtGRF9a*” and Potri.012G022600 as “*PtGRF9b*”; **Supplementary Figure S7**). *PtGRF9b* was not present in our set of high LOE genes because it has a breadth score of 2 and was not associated with any cell wall phenotypes through GWAS analyses. Because *PtGRF9b* had strong positive coexpression with *PtGRF9a* and shared edges with many cell wall genes, we included *PtGRF9b* in further analyses.

**Figure 4:**
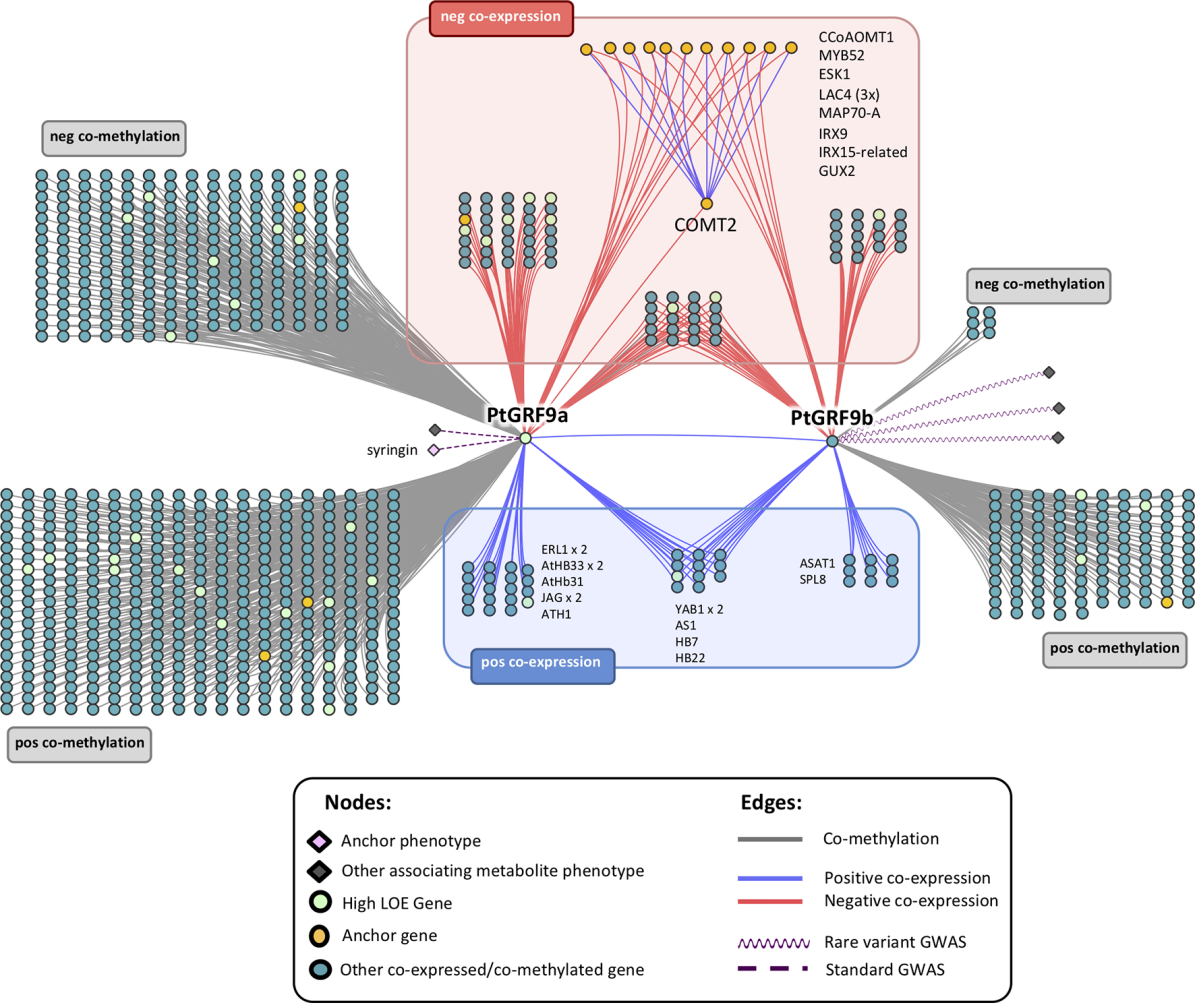
Genome-wide neighborhood of *PtGRF9* paralogs in the global input layer networks. Gene symbols are Arabidopsis Best-hit matches. See **Supplementary Table S10** for detailed node information; for functional enrichment details, see **Supplementary Tables S11** (negatively co-expressed genes) and S12 (positively co-expressed genes).

We constructed genome-wide 1-hop networks around each *PtGRF9* paralog across all data layers to assess the functional annotations of nearest neighbors (**Figure 4**; see **Supplementary Table S10** for detailed information about nodes). *PtIQD10* is present in the 1-hop network, along with many other genes with documented roles in cell wall processes. *PtGRF9a* and *PtGRF9b* are jointly positively co-expressed with 14 genes (one of which is a high LOE gene related to cell wall processes) and are jointly negatively co-expressed with 27 genes (including 7 cell wall anchor genes and 2 high LOE genes), implying an overlap in function. However, the bulk of neighbor genes are unique to each paralog, indicating divergence and perhaps specialization for specific tissues and conditions. GO-term functional enrichment analysis of the negative co-expression nodes in the 1-hop network showed significant enrichment for cell wall biological processes, including lignin biosynthesis, xylan biosynthesis and cell wall organization or biogenesis (**Supplementary Table S11**). In addition, the metabolite-GWAS association between *PtGRF9a* and syringin (a monolignol glucoside) indicated this SNP is associated with an allelic effect on syringin concentration (**Supplementary Figure S8**), further implicating *PtGRF9a* and *PtGRF9b* as repressors of secondary cell wall formation.

In Arabidopsis, *AtGRF7* is one of nine members of the *GRF* family of transcription factors (there are 20 *GRF* homologs in *P. trichocarpa*) that affect growth *via* multiple mechanisms (Omidbakhshfard et al., 2015). AtGRF7 has specifically been shown to modulate drought response by repressing *DREB2A* (Joshi et al., 2016) which ensures that drought response genes normally activated by DREB2A are not expressed under non-drought conditions, thus avoiding reduced growth. In addition to stress response, *GRF* genes are involved in regulating cell proliferation and differentiation in the shoot apical meristem (SAM). *GRF* genes therefore impact the elongation of stems, new leaf initiation, and the size and shape of leaves (Gonzalez et al., 2012). The phenotypic penetrance may occur as part of a complex formed with GRF Interacting Factor (GIF1/AN3) proteins (Hoe Kim and Tsukaya, 2015), where the GRF-GIF complex serves as a transcriptional activator, recruits chromatin remodeling complexes, and regulates the meristematic state of a tissue.

GO-term enrichment analysis of the positive coexpression nodes in the *PtGRF9* 1-hop network was consistent with roles reported in the literature for *GRF* genes (**Supplementary Table S12**). The most significantly enriched Biological Process GO terms include specification of axis polarity, shoot system development, shoot system morphogenesis and negative regulation of cell proliferation. Numerous osmotic-stress related genes are also found in the *PtGRF9* network (e.g., *AHA1/OST2*, *ERL1*, *PIP2;2*, *TIP4*;*1*, and *AREB3*), reflecting the well-documented relationship between *AtGRF7* and drought response. Significant connections between the *PtGRF9* paralogs and *PtGIF1* or *PtDREB2A* are not present in our LOE network. On closer inspection of co-expression values across tissues we see that *PtGRF9a* and *PtGIF1* do coexpress strongly in bud and immature leaf, but expression diverges in mature leaf and roots which causes the strength of coexpression to fall just below our 0.85 threshold (**Supplementary Figure S9**). The case is less clear for *PtDREB2A* as it shows little expression in most tissues.

Evidence that the *PtGRF9* paralogs play roles in regulating growth, defense, stress response, secondary growth, and cell wall biosynthesis suggest that *PtGRF9a* and *PtGRF9b* could be transcriptional co-regulators as described by Xie et al. (2018b), acting as master regulators that direct the global allocation of energy within a plant.

#### Evidence for Regulation of the Cell Wall by *PtGRF9*

To date, a role for the *GRF* family in cell wall regulation has not been reported, though it has been noted that cell proliferation and timing of differentiation must require control or delay of secondary cell wall deposition (Mele et al., 2003). Barros et al. (2015) noted that lignin cannot be removed once deposited, thus, specific regulatory mechanisms are required to control lignin biosynthesis and deposition at specific stages during cell differentiation. The contrasting patterns of coexpression between cell wall biosynthesis and meristematic control in our *PtGRF9* 1-hop network (**Figure 4**) suggest that it could be involved in such a mechanism. Furthermore, the GWAS association with syringin suggests that allelic variation in *PtGRF9a* in this population may have an additive effect on the amount of sinapyl alcohol stored or released for cell wall lignification.

Knowledge regarding downstream targets of *GRF* genes is incomplete (see Omidbakhshfard et al., 2015 for a comprehensive review). AtGRF7 has been shown to repress *AtDREB2A* by binding to the motif *TGTCAGG* (Kim et al., 2012). Additionally, the central *CAG* sub-motif is enriched in the promoter of *KNOX* genes that are targeted by *GRFs* (Kuijt et al., 2014). We searched for the complete *TGTCAGG* motif in the promoter regions of Arabidopsis homologs of the genes that coexpress with *PtGRF9a* using the online *athamap.de* tool, revealing two potential AtGRF7 targets in our 1-hop network: caffeoyl coenzyme A *O*-methyltransferase 1 (AT4G34050/*AtCCoAOMT1*) and MADS-box transcription factor *AtAGL12* (AT1G71692). Both genes are relevant to the cell wall, and *P. trichocarpa* homologs of these genes are negatively co-expressed with *PtGRF9a*. To further investigate these genes as potential PtGRF9a targets, we used Analysis of Motif Enrichment (AME) (McLeay and Bailey, 2010), but found no evidence for enrichment of the *TGTCAGG* motif in the 2-kb upstream or CDS regions of *PtCCoAOMT* (Potri.001G304800 and Potri.009G099800) or *PtAGL12* (Potri.013G102600). Manual examination revealed that the *TGTCAGG* motif appears inexactly in the upstream regions of *PtCCoAOMT1* and *PtAGL12* (*TGTTCAGG* in *CCoAOMT1* Potri.009G099800; *TGTCAGC* in *PtCCoAOMT* Potri.001G304800 and *PtAGL12*). Consistent with the findings of Franco-Zorrilla et al. (2014), who show that repressor TFs such as PtGRF9a are more likely than activator TFs to bind downstream of a target gene, we found 27 *Populus* genes significantly enriched for *TGTCAGG* in the 1-kb downstream region, including *PtMYB41* (Potri.012G039400, a homolog of *AtMYB52*), which is negatively coexpressed with *PtGRF9a*. AtMYB52 is a TF known to induce secondary cell wall biosynthesis genes and its repression reduces secondary wall thickening in fibers (Zhong et al., 2008). Furthermore, *AtMYB52* overexpression has been linked with drought tolerance (Park et al., 2011). Given the established role of AtGRF7 in drought response, repression of *PtMYB41* is a potential avenue for PtGRF9a to regulate both lignification and drought tolerance, although further experimental evidence is required.

**Figure 5:**
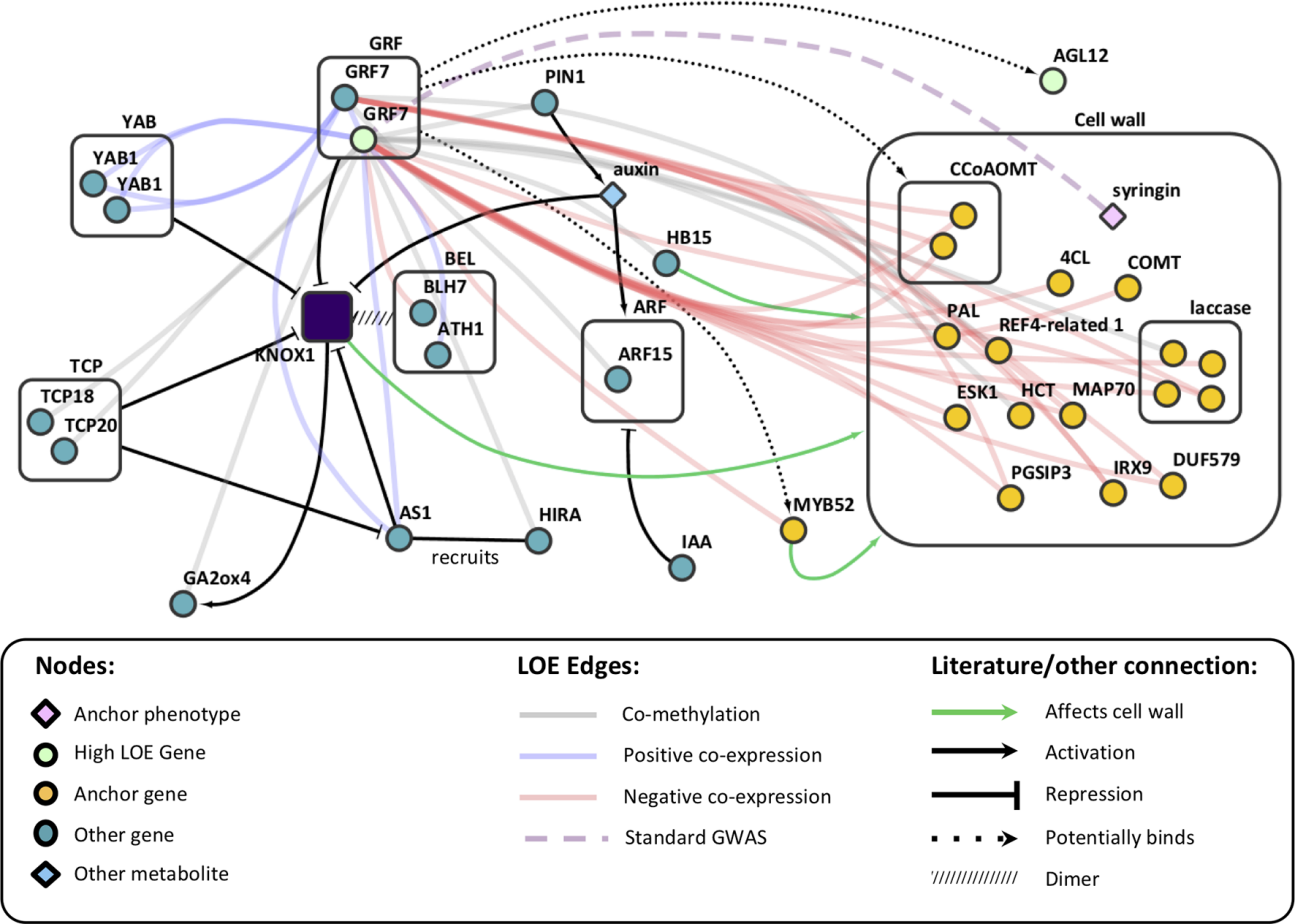
Literature evidence linking *AtGRF7* genes to *AtKNOX* genes and the cell wall, overlaid on LOE edges from the *PtGRF9* subnetwork. See **Supplementary Table S13** for *P. trichocarpa* orthologs.

Analysis of our 1-hop network suggests that *PtGRF9* also affects cell wall biosynthesis by regulating a host of homeobox genes. Twenty homeobox genes were present in the *PtGRF9* network, including *PtATHB.12* (Potri.001G188800; homolog of *AtHB15*/AT1G52150), which has been shown to influence secondary wall formation and cambial production of xylem (Schrader, 2004; Cassan-Wang et al., 2013), and *PtAGL12* (Du et al., 2009; Du et al., 2011; Weighill et al., 2018) (**Supplementary Figure S5**). There was also indirect evidence in the *PtGRF9* network suggesting PtGRF9 interacts with *PtKNOX* genes. *KNOX* genes are involved in meristem maintenance and are downregulated to facilitate lateral primordia development and the differentiation of cambium into xylem and phloem (Hertzberg et al., 2001; Schrader, 2004; Hay and Tsiantis, 2010). *GRF* genes are involved in specification of primordia cells and have been shown to repress *KNOX* genes by forming hairpins in targeted regions (Kuijt et al., 2014; Hoe Kim and Tsukaya, 2015). Interactions between AtGRF7 and *KNOX* genes have yet to be investigated, but the primary motif of the target sequence by which AtGRF7 binds *AtDREB2A* was shown to be enriched in several *KNOX* genes, and experiments in rice, barley, and Arabidopsis have confirmed that multiple *GRF* genes bind these motifs in *KNOX* genes (Kim et al., 2012; Kuijt et al., 2014). The presence of several genes that exclusively or directly interact with *KNOX* genes in the 1-hop network strongly implies that PtGRF9 proteins influence the cell wall *via* interactions with the *PtKNOX1* genes *PtSTM* and *PtBP*, and likely other *PtKNOX* genes as well (**Supplementary Table S13** and **Figure 5**). Although *KNOX* family genes were not present in the *PtGRF9* network, this was likely due to highly tissue-specific expression patterns which our coexpression analysis methods were not designed to detect (see **Supplementary Figure S10**).

The *PtKNOX*-associated genes in the *PtGRF9* network have documented roles in cell wall and secondary growth phenotypes (**Figure 5**). SHOOT-MERISTEMLESS (PtSTM) downregulates gibberellic acid levels by repressing gibberellin 20-oxidase (*PtGA20ox*) biosynthesis genes and upregulating catabolism genes such as *PtGA2ox4* (positively co-methylated with *PtGRF9a*), which inhibits xylem production (Eriksson et al., 2000; Jasinski et al., 2005). Overexpression of *PtSTM*/ARBORKNOX1 (*PtSTM*/*PtARK1*) in *P. tremula* × *P. alba* has been shown to inhibit differentiation of leaf primordia, elongation of internodes, and differentiation of secondary vascular cells (Groover et al., 2006). Counterintuitively, overexpression of *PtSTM/PtARK1* in secondary meristems also results in upregulation of some lignin biosynthesis genes and increased lignin content. Long-term transcriptional repression of BREVIPEDICELLUS (*AtBP*), KNOTTED-like 2 from *A. thaliana* (*AtKNAT2*) and *AtKNAT6* outside the meristem is facilitated by chromatin remodeling carried out by the protein encoded by ASYMMETRIC LEAVES 1 (*AtAS1*; *PtAS1* is positively co-expressed with *PtGRF9a* and *PtGRF9b*), which dimerizes with AtAS2 and recruits the histone chaperone protein encoded by *AtHIRA* (*PtHIRA* is negatively co-methylated with *PtGRF9a*) (Guo et al., 2008; Hay and Tsiantis, 2010). AS2 is involved in controlling seasonal lignification in spruce, likely through its role in repressing *BP* (Jokipii-Lukkari et al., 2018). BP decreases lignin deposition and regulates the localization of lignification by binding the promoters of *AtCOMT1*, *AtCCoAOMT1*, laccases, and peroxidases (putative orthologs of which are all negatively co-expressed with *PtGRF9a* and *PtGRF9b*) (Mele et al., 2003). The *PtGRF9* network includes many of the cell wall biosynthesis-related genes that Mele et al. (2003) found to be differentially expressed in *bp* mutants, including five putative orthologs (*PAL1*, *OMT1*, two *CCoAOMT1* paralogs, *PME3*, and *GH9B5*; see **Supplementary Table S13**) and an additional 23 genes belonging to the same families as differentially expressed genes in *bp* mutants (*4CL2*, five *PME*s, *KCS19*, four peroxidases, four laccases, *ERD4*, *GAUT4*, *PUB24*, *MEE23*, *ERF1-3*, and three R2R3 MYBs: *MYB52*, *MYB93*, *MYB111*). Consistent with these observations in Arabidopsis, overexpression of *AtBP*/ARBORKNOX2 (*AtBP/AtARK2*) in *P. alba* x *P. tremula* results in downregulation of ABNORMAL FLORAL ORGANS (*PtAFO/PtYAB1*), PIN-FORMED 1 (*PtPIN1*), *PtAGL12* (all negatively co-expressed with *PtGRF9a*) and *PtGA20ox* genes, leading to inhibition of cellular differentiation and division and decreases in biomass (Du et al., 2009). Furthermore, overexpression of *PtBP/PtARK2* results in downregulation of cell wall biosynthesis genes, decreased lignin content, reduced phloem fibers, and reduced secondary xylem in stems.

We did not find a connection between the *PtGRF9* genes and cell wall anchor genes *KNAT7* (Potri.001G112200, a *PtKNOX2* gene) and BEL1-like homeodomain 6 genes (*PtBLH6*, Potri.004G159300 and Potri.009G120800). These genes have well-documented roles in cell wall regulation (Li et al., 2012a; Cassan-Wang et al., 2013). However, the *PtGRF9* genes do not appear to be involved in their regulation, perhaps because *PtKNOX2* genes are generally more functionally diverse and broadly expressed than *PtKNOX1* genes (consistent with expression data in **Supplementary Figure S10**) (Furumizu et al., 2015). In addition, they are not involved in meristematic maintenance, and in some cases seem to overlap in function with genes that promote differentiation (consistent with what we recovered in our LOE analysis).

#### *PtGRF9* eQTN Network: An Independent Line of Evidence

**Figure 6:**
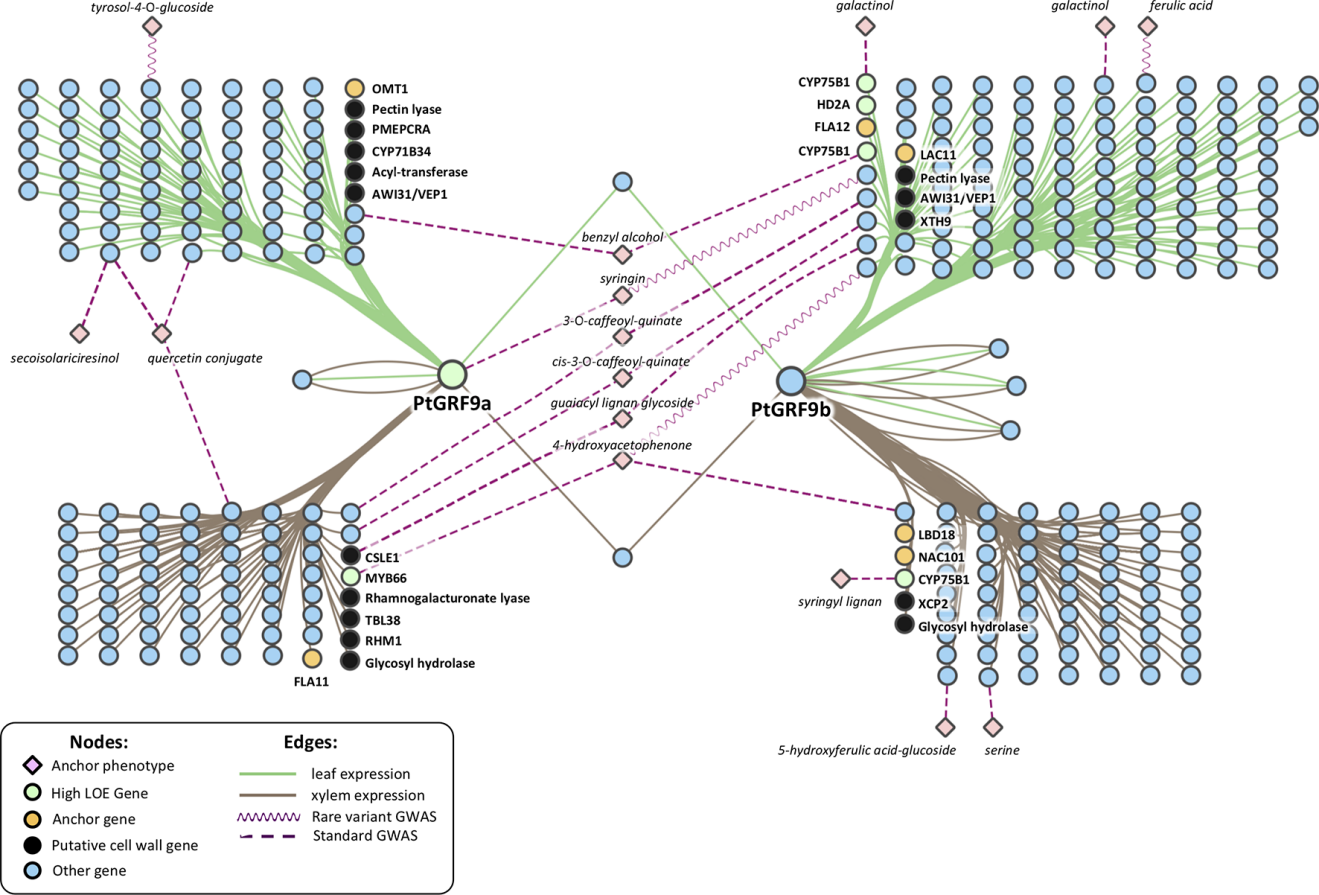
Two-hop network created by merging a 1-hop eQTN network around the *PtGRF9* paralogs and 1-hop metabolite-GWAS networks around anchor metabolites. See **Supplementary Table S14** for node information.

As a means of independently evaluating support for the hypothesis that *PtGRF9* paralogs regulate cell wall biosynthesis, we constructed 1-hop eQTN networks around *PtGRF9a* and *PtGRF9b* (**Figure 6**; detailed node information available in **Supplementary Table S14**). SNPs in both the *PtGRF9a* and *PtGRF9b* 1-hop networks were associated with cell wall expression phenotypes in leaf and xylem tissues, as well as expression phenotypes consistent with the previously documented roles of *AtGRF7* and other *GRF* orthologs in regulating functions such as growth, defense, and stress response. In agreement with the multi-omic 1-hop network described in the section *GROWTH-REGULATING FACTOR 9: Putative Master Regulator* (**Figure 4**), the eQTN network indicated each paralog has connectivity with cell-wall-related genes affecting multiple facets of cell wall biosynthesis, including transcriptional regulation, cellulose biosynthesis, lignin biosynthesis, xylan biosynthesis, and secondary cell wall deposition. Also consistent with the multi-omic 1-hop network, the eQTN analysis indicated that despite a low degree of topological overlap between the *PtGRF9a* and *PtGRF9b* neighborhoods, the paralogs still largely overlap in function.

To gain an understanding of how the *PtGRF9* paralogs potentially affect cell wall metabolites, the 1-hop eQTN network was merged with 1-hop anchor metabolite networks generated from traditional and rare variant metabolite-GWAS data layers. Beyond the direct GWAS association of *PtGRF9a* with syringin, 14 additional anchor metabolites are present in the 2-hop eQTN to metabolite-GWAS network (**Figure 6**), 6 of which are indirectly associated with both paralogs through various intermediate genes. There appears to be a pattern of segregation regarding metabolite associations between tissue types and *PtGRF9* paralogs, perhaps indicating that these genes are diverging to fulfill different tissue-specific regulatory roles.

### Future Directions

Our extended network analysis pipeline has provided a short list of putative cell wall regulatory genes to the scientific community for experimental validation. We performed an in-depth investigation of the *PtGRF9* paralogs, which are particularly promising candidates for regulation of cell wall biosynthesis and secondary growth. Furthermore, we show the *PtGRF9* paralogs are potential transcriptional co-regulators that coordinate the flow of energy among growth, defense, stress response, and lignification, in a manner consistent with the hypothesis of Xie et al. (2018b). The ability to manipulate transcriptional co-regulators such as these *via* genetic engineering and breeding programs would provide a powerful tool for shaping bioenergy crops.

Incorporating a rare variant metabolite-GWAS data layer in the LOE analysis has proven to be a valuable asset in the identification of new candidate genes. Incorporating a genome-wide eQTN (SNP-to-expression-phenotype GWAS) data layer in future analyses would provide greater clarity regarding the mechanisms through which these genes regulate cell-wall-related functions. Furthermore, DNA affinity purification sequencing (DAP-seq) could provide further support for hypothesized transcription factor binding sites, and thus help elucidate relevant transcription factor regulatory networks. Tissue-specific expression analysis across a GWAS population would allow for increased “tissue level resolution” of the regulatory networks. The extended network analysis pipeline will be a valuable tool to integrate these new layers with the previous networks to produce a holistic model of cell wall regulation.

## Data Availability Statement

*Populus trichocarpa* genome sequence, annotation, and Gene Atlas expression data sets are available on Phytozome (http://phytozome.jgi.doe.gov). *P. trichocarpa* variant data (DOI 10.13139/OLCF/1411410) is available from https://doi.ccs.ornl.gov/ui/doi/55. Scripts used to calculate LOE scores, create GO-term networks, and calculate weighted intersect scores are available on GitHub: https://github.com/afurches/Cell_Wall_LOE.

## Author Contributions

XR and RD provided anchor gene IDs. TT provided anchor metabolite IDs. WJ developed the Parallel GPU CCC application code. DK performed the rare variant GWAS analysis. PJ performed the standard GWAS and outlier analysis and constructed the standard GWAS and eQTN networks. DW calculated methylation TPM values and constructed the observed co-expression, co-methylation and SNP correlation networks and calculated LOE scores for the observed LOE network. AF performed expression and methylation randomizations. JG performed random subsampling. AF calculated correlation values and performed significance tests and performed LOE input layer randomizations, constructed randomized LOE networks, and calculated randomized LOE scores. DK and JR created the GO-term network, and AF calculated GO intersect scores. AF performed candidate ranking, generated the clustered expression heatmap, and performed the phylogenetic analyses. DK performed the functional enrichment and TF-binding analyses. AF created eQTN subnetworks. MS mapped gene expression atlas reads and RNAseq reads and calculated gene expression TPM values. SD and GT led the effort on constructing the GWAS population. TT led the leaf sample collection for GCMS-based metabolomic analysis, identified the peaks, and summarized the metabolomics data. PR did automated extraction of metabolite intensity from GCMS. MM collected the leaf samples and manually extracted the metabolite data. NZ performed leaf sample preparation, extracted, derivatized, and analyzed the metabolites by GCMS. JSch and AS generated the gene expression atlas data. SD and DM-S generated the SNP calls. J-GC provided RNAseq data. AF, AW, DK, and AL performed the in-depth literature searches. AL performed the domain annotation. AF, DK, DW, AL, and JStr wrote the manuscript. All authors edited the manuscript. DJ conceived of and supervised the study, participated in the network analysis, and generated MapMan annotations.

## Funding

Funding was provided by The Center for Bioenergy Innovation (CBI), U.S. Department of Energy Bioenergy Research Centers supported by the Office of Biological and Environmental Research in the DOE Office of Science.

An award of computer time was provided by the INCITE program. This research used resources of the Oak Ridge Leadership Computing Facility, which is a DOE Office of Science User Facility supported under Contract DE-AC05-00OR22725. This research also used resources of the Compute and Data Environment for Science (CADES) at the Oak Ridge National Laboratory, which is supported by the Office of Science of the U.S. Department of Energy under Contract No. DE- AC05-00OR22725.

Support for the Poplar GWAS dataset was provided by The BioEnergy Science Center (BESC) and The Center for Bioenergy Innovation (CBI). U.S. Department of Energy Bioenergy Research Centers supported by the Office of Biological and Environmental Research in the DOE Office of Science. The Poplar GWAS Project used resources of the Oak Ridge Leadership Computing Facility and the Compute and Data Environment for Science at Oak Ridge National Laboratory, which is supported by the Office of Science of the U.S. Department of Energy under Contract No. DE-AC05-00OR22725.

The JGI Plant Gene Atlas project conducted by the U.S. Department of Energy Joint Genome Institute was supported by the Office of Science of the U.S. Department of Energy under Contract No. DE-AC02-05CH11231.

AL and AW acknowledge that this project was supported in part by appointments to the Higher Education Research Experiences (HERE) Program at Oak Ridge National Laboratory, administered by the Oak Ridge Institute for Science and Education (ORISE). ORISE is managed by Oak Ridge Associated Universities (ORAU) for the U.S. Department of Energy (DOE).

The work conducted by the U.S. Department of Energy Joint Genome Institute is supported by the Office of Science of the U.S. Department of Energy under Contract No. DE-AC02-05CH11231.

## Conflict of Interest

The authors declare that the research was conducted in the absence of any commercial or financial relationships that could be construed as a potential conflict of interest.
