# Supplementary material for: Finding New Cell Wall Regulatory Genes in Populus trichocarpa Using Multiple Lines of Evidence
Source: Front Plant Sci. 2019 Oct 8;10:1249. doi: 10.3389/fpls.2019.01249 (PMC6791931; doi:10.3389/fpls.2019.01249)
Supplement: Supplementary file 1 [file Table_1.docx]

Supplementary Material

Finding New Cell Wall Regulatory Genes in *Populus trichocarpa* Using Multiple Lines of Evidence

**Anna Furches, David Kainer, Deborah Weighill, Annabel Large, Piet Jones, Angelica M Walker, Jonathon Romero, Joao Gabriel Felipe Machado Gazolla, Wayne Joubert, Manesh Shah, Jared Streich, Priya Ranjan, Jeremy Schmutz, Avinash Sreedasyam, David Macaya-Sanz, Nan Zhao, Madhavi Z. Martin, Xiaolan Rao, Richard A. Dixon, Stephen DiFazio, Timothy J. Tschaplinski, Jin-Gui Chen, Gerald A. Tuskan, Daniel Jacobson**

***Correspondence:**
Daniel Jacobson
jacobsonda@ornl.gov

# Supplementary Text S1: High LOE gene AGAMOUS-LIKE 12

There is evidence that high LOE gene AGAMOUS-LIKE 12 (*PtAGL12*, Potri.013G102600) functions in a regulatory role in cell wall biosynthesis regulatory role is MADS box transcription factor (Supplementary Table S4). *PtAGL12* is significantly associated with cis-3-*O*-caffeoyl-quinate via traditional metabolite-GWAS and with fructose via rare variant metabolite-GWAS (Supplementary Fig. S3) and is significantly coexpressed and comethylated with 20 cell wall anchor genes (Supplementary Table S6). AGAMOUS-LIKE genes form a large and diverse family, with many playing roles in floral organ identity, flowering time, and transition between reproductive and vegetative growth phases (Fernandez et al., 2014; Lee et al., 2000; Masiero et al., 2011; Ng and Yanofsky, 2001). There is also growing evidence that AGAMOUS-LIKE genes affect lignin content. A patent exists for altering the expression of *AtAGL8* to control lignin content in Arabidopsis (Yanofsky et al., 2004), and *AtAGL15* has been shown to regulate *PRX17*, which in turn affects lignin content (Cosio et al., 2017). In tomato, RNAi-silencing of *TAGL1* results in increased expression of lignin biosynthesis genes and lignin content (Giménez et al., 2010). Phylogenetic analysis indicates *PtAGL12* and Arabidopsis *AtAGL12* / AT1G71692 are orthologous (Supplementary Fig. S4); birch *BpMADS12,* Arabidopsis *AtAGL12*, and *PtAGL12* are SOC1/TM3 class MADS-box genes, which belong to the MIKC^C^-type of the type II MADS-box genes (Leseberg et al., 2006; Li et al., 2016). Li et al. (2016) found that lines overexpressing the *BpMADS12* gene had significantly higher lignin content than wild-type lines, suggesting *BpMADS12* might play a role in regulating the expression of lignin biosynthesis genes, possibly via brassinosteroid signalling.

# Supplementary Figures

Figure S1: (A) Distribution of observed gene coexpression correlation values (red) versus correlation values for randomized expression data (blue). We applied a threshold to exclude correlation values below 0.85 prior to LOE analysis (threshold indicated by vertical red line). (B) Distribution of observed gene comethylation correlation values (red) versus correlation values for randomized methylation data (blue). We applied a threshold to exclude correlation values below 0.95 prior to LOE analysis (threshold indicated by the vertical red line).

**2A)**

**Breadth Score Distribution: All genes**

**2B)**

Figure S2: A) Distribution of LOE breadth scores for all genes. B) Distributions of LOE scores for high LOE genes.

Figure S3: MapMan annotations of high-LOE genes.

Figure S4: LOE subnetwork depicting the one-hop neighbors of *PtIQD10* (Potri.011G096500). Gene symbols are Arabidopsis Best-hit matches. See Supplementary Table S7 for detailed node information.

Figure S5: LOE subnetwork depicting the one-hop neighbors of *P. trichocarpa* *PtAGL12* (Potri.013G102600). Gene symbols are Arabidopsis Best-hit matches. See Supplementary Table S8 for detailed node information.


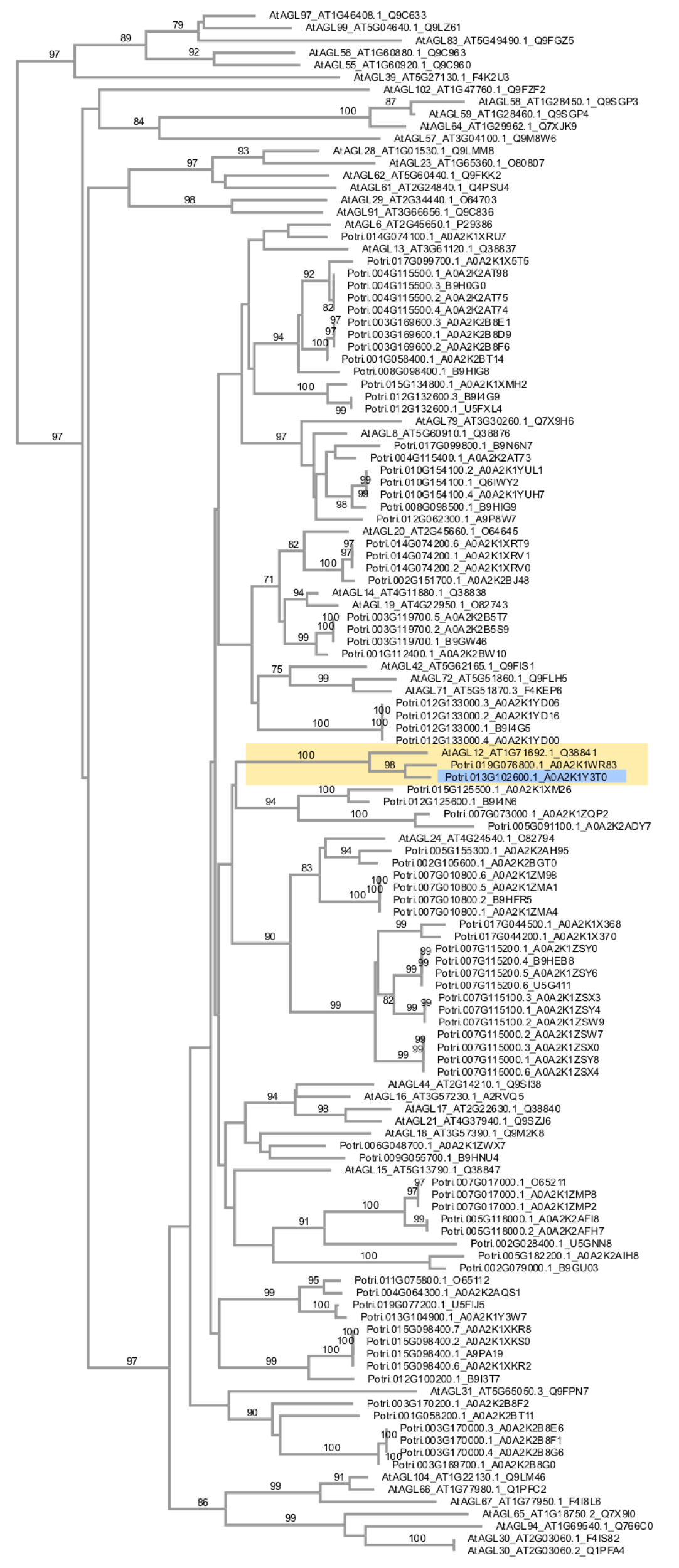


Figure S6: Phylogram of *Populus trichocarpa* and *Arabidopsis thaliana* AGAMOUS-LIKE (AGL) amino acid sequences. *PtAGL12* (Potri.013G102600; indicated in blue) is a member of the clade containing *AtAGL12* (AT1G71692; in yellow). To assess support for orthology, we performed reciprocal BLASTp searches of amino acid sequences containing the SRF-type transcription factor domain (PF00319) and K-box region domain (PF01486) from A. thaliana and P. trichocarpa (obtained from UniProt; www.uniprot.org) and a phylogenetic analysis. Canonical amino acid sequences containing the SRF-type transcription factor (PF00319) domain and K-box region (PF01486) domain for Arabidopsis and *P. trichocarpa* were obtained from UniProt (www.uniprot.org). *AGL* amino acid sequences were aligned using MAFFT v7 (Katoh et al., 2017; Kuraku et al., 2013) using the E-INS-i method, BLOSUM62 scoring matrix, gap opening penalty of 1.53, offset value of 0.0, and default guide tree settings. A UPGMA tree was calculated to assess high-level relationships, in which sequences were grouped into two large clades. Sequences belonging to the same clade as *AtAGL12* were retained (which included all *P. trichocarpa* sequences) and a new alignment was created using the same parameters described above. A neighbor-joining tree was constructed from the second alignment using all “gap-free sites” (84 amino acids), a Poisson substitution model, and bootstrapping with 1000 replicates. A phylogram was created using Archaeopteryx.js (Han and Zmasek, 2009; Zmasek and Eddy, 2001). Bootstrap scores above 70 are indicated on nodes. Our results support an orthologous relationship between PtAGL12 and AtAGL12, which is consistent with the phylogenetic analysis of (Leseberg et al., 2006).


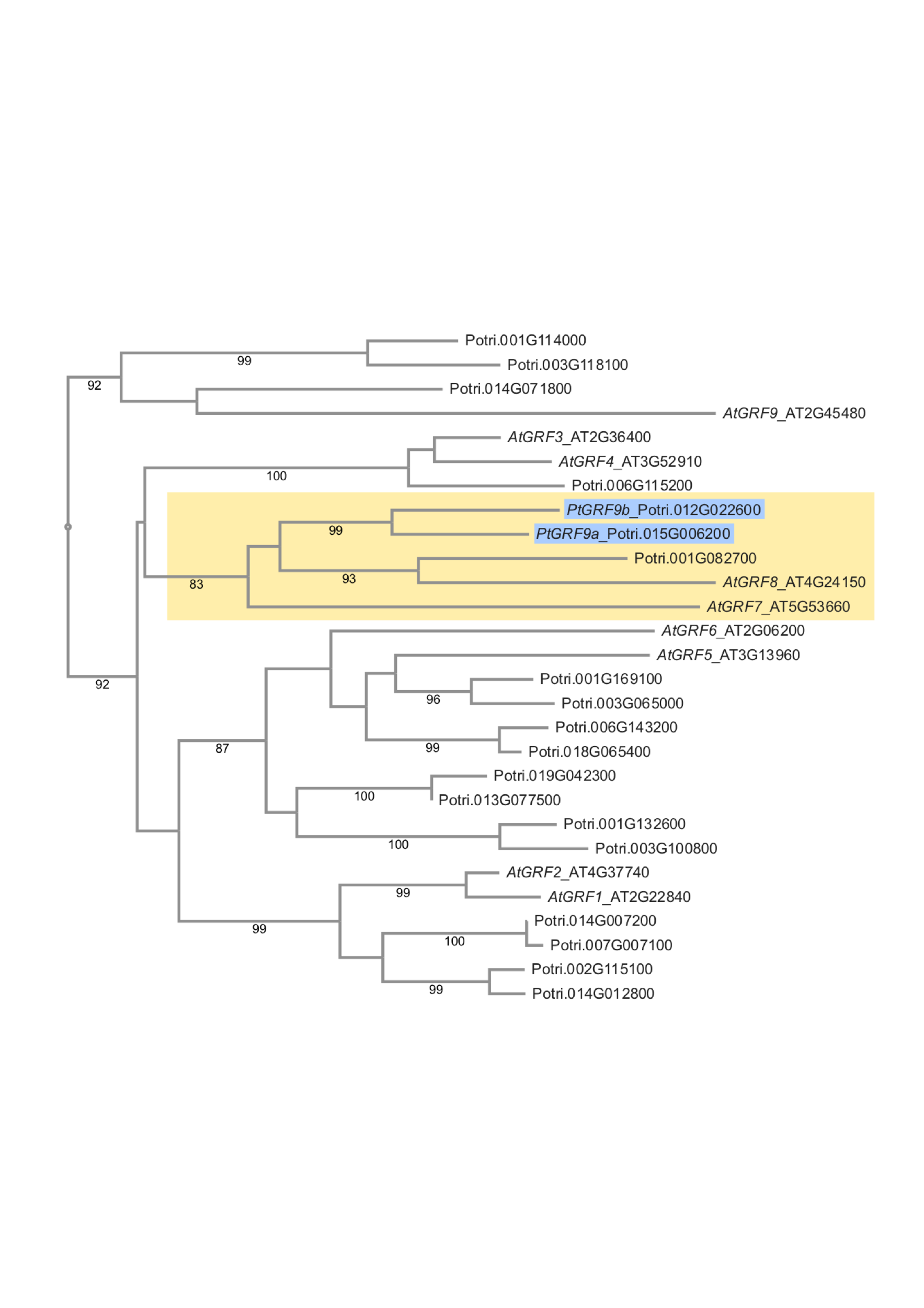


Figure S7: Phylogram of *Arabidopsis thaliana* and *Populus trichocarpa* GROWTH-REGULATING FACTOR (*GRF*) amino acid sequences. *PtGRF9a* and *PtGRF9b* (indicated in blue) belong to the clade containing *AtGRF7* (in yellow). Canonical amino acid sequences containing the WRC (PF08879) and QLQ (PF08880) domains for Arabidopsis and *P. trichocarpa* were obtained from UniProt (www.uniprot.org). GRF proteins were aligned using MAFFT v7 (Katoh et al., 2017; Kuraku et al., 2013) using the E-INS-i method, BLOSUM62 scoring matrix, gap opening penalty of 1.53, offset value of 0.0, default guide tree settings, MAFFT-homologs option with 50 homologs and a threshold of E=1e-10. A neighbor-joining tree was constructed using all “gap-free sites” (92 amino acids), a Poisson substitution model, and bootstrapping with 1000 replicates. A phylogram was created using Archaeopteryx.js (Han and Zmasek, 2009; Zmasek and Eddy, 2001). Bootstrap scores above 70 are indicated on nodes.

.
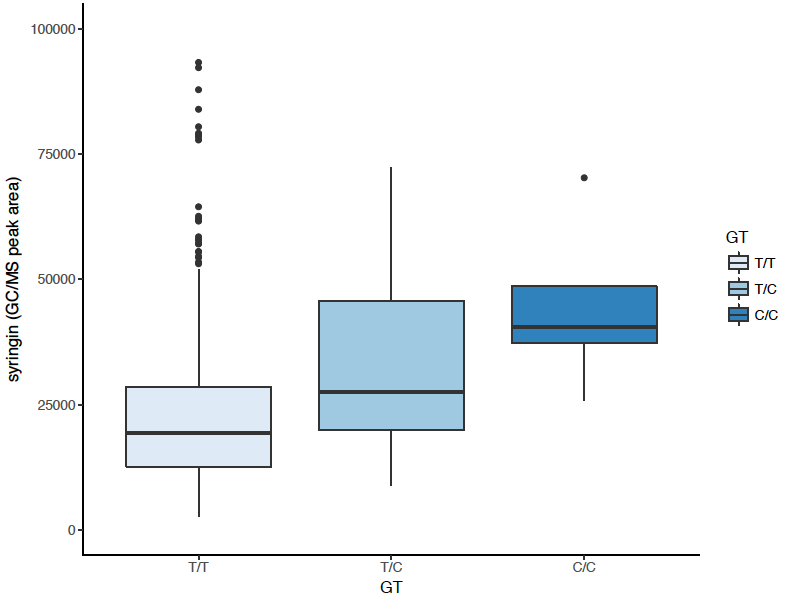


Figure S8: Allelic effect of the SNP located 3,788bp upstream of the *PtGRF9a* coding region that associates with syringin. SNP location: Chr15:400369; *PtGRF9a* location: Chr15:394635..396581 (reverse strand).


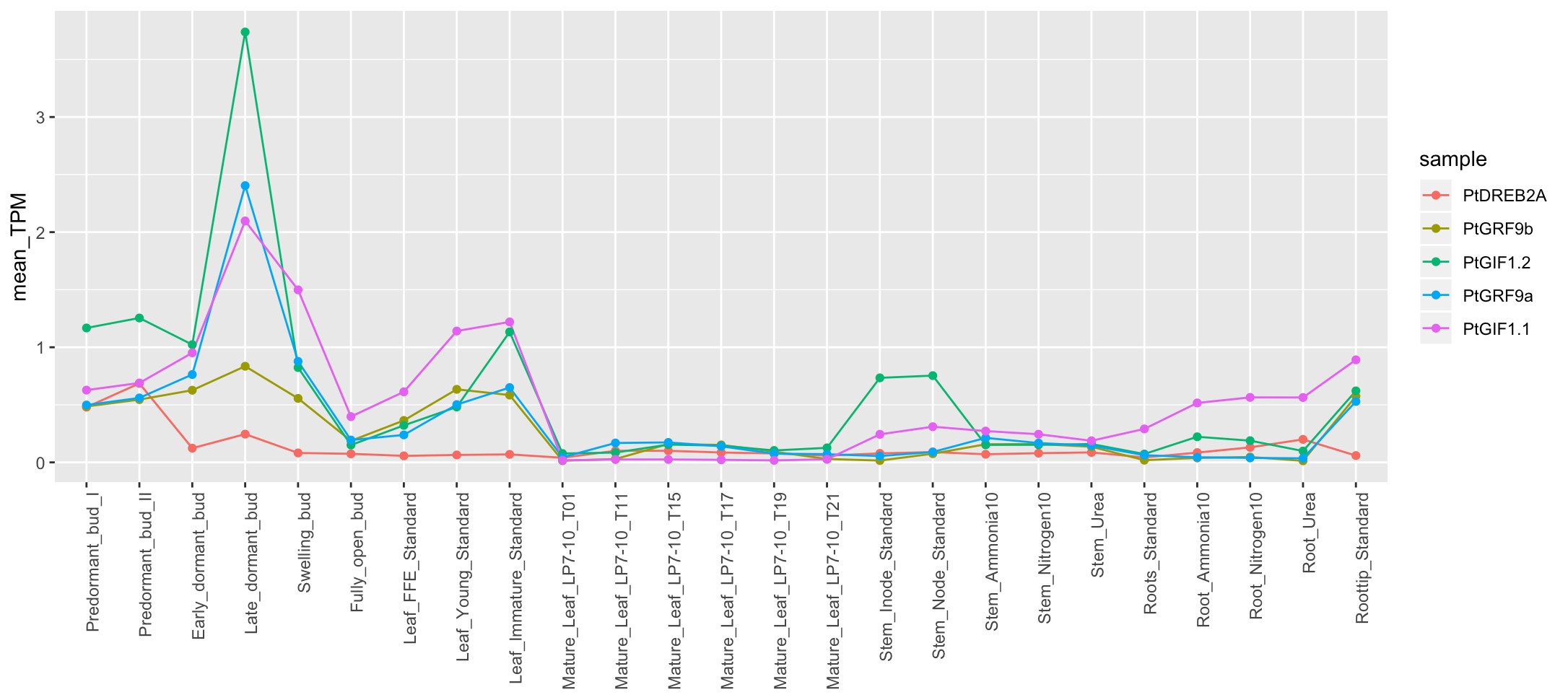


Figure S9: Correlation between *P. trichocarpa* *PtGRF9a, PtGRFb, PtGIF1* and *PtDREB2A*. See Supplemental Table S6 for additional information about Gene Atlas expression data.

Figure S10: Tissue-specific expression heatmap of *P. trichocarpa* *PtGRF9* paralogs (indicated by black pentagons), all *PtKNOX genes*, all *PtBELL genes*, and a subset of genes present in the *PtGRF9* 1-hop network: putative orthologs of *AtKNOX*-associated genes and cell wall biosynthesis genes down-regulated in *bp* mutants (Mele et al., 2003) (indicated by white pentagons; additional details are listed in Supplementary Table S13). Genes names with asterisks (*) do not have gene symbols or aliases in the *Populus trichocarpa* v3.0 genome annotation (Phytozome), therefore gene symbols from Arabidopsis Best-hit matches were used. See Supplementary Table S1 for additional information about Gene Atlas tissue samples.

# Supplementary Tables

Table S1: Additional information about Gene Atlas expression data.

Table S2: Cell wall anchor genes used as input for LOE analysis.

Table S3: Cell wall anchor metabolites used as input for LOE analysis.

Table S4: Number of high LOE genes with regulatory-like MapMan annotations.

Table S5: High LOE genes with documented cell wall-related roles, including anchor genes retrieved as high LOE genes.

Table S6: LOE scores, Priority Tiers, and functional annotations for ranked and filtered high LOE genes (metabolite-GWAS score ≥1, breadth score ≥3).

Table S7: Node information for Supplemental Fig. S2 (one-hop LOE subnetwork surrounding *P. trichocarpa* *PtIQD10*).

Table S8: Node information for Supplementary Fig. S4 (one-hop LOE subnetwork around *P. trichocarpa* *PtAGL12*).

Table S9: Additional information for genes in Table 1 (Tier 1 of ranked high LOE genes).

Table S10: Node information for Figure 5 (Genome-wide neighborhood of P. trichocarpa PtGRF9 paralogs).

Table S11 BinGO output for genes negatively co-expressed with *PtGRF9* paralogs.

Table S12: BinGO output for genes positively co-expressed with *PtGRF9* paralogs.

Table S13: KNOX-associated genes and differentially expressed genes in bp mutants (Mele et al., 2003) that are present in the PtGRF9 genome-wide 1-hop network.

Table S14: Nodes present in the eQTN one-hop network surrounding *PtGRF9* paralogs.

# References

Cosio, C., Ranocha, P., Francoz, E., Burlat, V., Zheng, Y., Perry, S. E., et al. (2017). The class III peroxidase PRX17 is a direct target of the MADS-box transcription factor AGAMOUS-LIKE15 (AGL15) and participates in lignified tissue formation. New Phytol. 213, 250–263. doi:10.1111/nph.14127.

Fernandez, D. E., Wang, C.-T., Zheng, Y., Adamczyk, B. J., Singhal, R., Hall, P. K., et al. (2014). The MADS-Domain Factors AGAMOUS-LIKE15 and AGAMOUS-LIKE18, along with SHORT VEGETATIVE PHASE and AGAMOUS-LIKE24, Are Necessary to Block Floral Gene Expression during the Vegetative Phase. Plant Physiol. 165, 1591–1603. doi:10.1104/pp.114.242990.

Giménez, E., Pineda, B., Capel, J., Antón, M. T., Atarés, A., Pérez-Martín, F., et al. (2010). Functional analysis of the Arlequin mutant corroborates the essential role of the ARLEQUIN/TAGL1 gene during reproductive development of tomato. PLoS One 5. doi:10.1371/journal.pone.0014427.

Han, M. V, and Zmasek, C. M. (2009). phyloXML: XML for evolutionary biology and comparative genomics. *BMC Bioinformatics* 10, 356. doi:10.1186/1471-2105-10-356.

Katoh, K., Rozewicki, J., and Yamada, K. D. (2017). MAFFT online service: multiple sequence alignment, interactive sequence choice and visualization. *Brief. Bioinform.* doi:10.1093/bib/bbx108.

Kuraku, S., Zmasek, C. M., Nishimura, O., and Katoh, K. (2013). aLeaves facilitates on-demand exploration of metazoan gene family trees on MAFFT sequence alignment server with enhanced interactivity. *Nucleic Acids Res.* 41, W22–W28. doi:10.1093/nar/gkt389.

Lee, H., Suh, S. S., Park, E., Cho, E., Ahn, J. H., Kim, S. G., et al. (2000). The AGAMOUS-lIKE 20 MADS domain protein integrates floral inductive pathways in Arabidopsis. Genes Dev. 14, 2366–2376. doi:10.1101/gad.813600.

Leseberg, C. H., Li, A., Kang, H., Duvall, M., and Mao, L. (2006). Genome-wide analysis of the MADS-box gene family in Populus trichocarpa. Gene 378, 84–94. doi:10.1016/j.gene.2006.05.022.

Li, H., Yang, Y., Wang, Z., Guo, X., Liu, F., Jiang, J., et al. (2016). BpMADS12 gene role in lignin biosynthesis of Betula platyphylla Suk by transcriptome analysis. J. For. Res. 27, 1111–1120. doi:10.1007/s11676-016-0229-y.

Masiero, S., Colombo, L., Grini, P. E., Schnittger, A., and Kater, M. M. (2011). The Emerging Importance of Type I MADS Box Transcription Factors for Plant Reproduction. Plant Cell 23, 865–872. doi:10.1105/tpc.110.081737.

Ng, M., and Yanofsky, M. F. (2001). Function and evolution of the plant MADS-box gene family. Nat Rev Genet 2, 186–195. doi:10.1038/35056041.

Zmasek, C. M., and Eddy, S. R. (2001). ATV: display and manipulation of annotated phylogenetic trees. *Bioinformatics* 17, 383–4. Available at: http://www.ncbi.nlm.nih.gov/pubmed/11301314.
